# Evaluation of automatic cell free DNA extraction metrics using different blood collection tubes

**DOI:** 10.1038/s41598-025-03508-4

**Published:** 2025-06-03

**Authors:** Daniel Andersson, Helena Kristiansson, Manuel Luna Santamaría, Huma Zafar, Ivan Mijakovic, Åsa Torinsson Naluai, Anders Ståhlberg

**Affiliations:** 1 Sahlgrenska Center for Cancer Research, Department of Laboratory Medicine, Institute of Biomedicine, Sahlgrenska Academy, University of Gothenburg, 413 90 Gothenburg, Sweden; 2 Department of Clinical Genetics and Genomics, Sahlgrenska University Hospital, 413 45 Gothenburg, Sweden; 3 Wallenberg Centre for Molecular and Translational Medicine, University of Gothenburg, 413 90 Gothenburg, Sweden; 4 Biobank West, Sahlgrenska University Hospital, 413 45 Gothenburg, Sweden; 5 Division of Systems and Synthetic Biology, Department of Life Sciences, Chalmers University of Technology, 412 96 Gothenburg, Sweden; 6 The Novo Nordisk Foundation Center for Biosustainability, Technical University of Denmark, 2800 Kongens Lyngby, Denmark; 7 Department of Laboratory Medicine, Institute of Biomedicine and Core Facilities, Sahlgrenska Academy, University of Gothenburg, 405 30 Gothenburg, Sweden; 8 SciLifeLab, Institute of Biomedicine, University of Gothenburg, 413 90 Gothenburg, Sweden

**Keywords:** Automated extraction, Cell-free DNA, Pre-analytics, Liquid biopsy, Blood plasma, Molecular medicine, Diagnostic markers, Biomarkers

## Abstract

Liquid biopsies and cell-free DNA (cfDNA) analysis are used in numerous clinical applications. The amount of cfDNA is generally limited and many approaches require assessment of individual molecules. Optimized pre-analytical steps are therefore fundamental for accurate interpretation. Here, we established an automated extraction approach providing cfDNA of high yield and quality. We analyzed 649 blood plasma samples collected from 23 healthy individuals and assessed the performance of four different blood collection tubes, time between sampling and plasma isolation and number of centrifugation steps. CfDNA was quantified by fluorometric analysis and quantitative polymerase chain reaction, while contaminating cellular DNA was assessed by quantitative polymerase chain reaction and parallel capillary electrophoresis. Data showed that cfDNA yield depends on both choice of blood collection tube and time between sampling and plasma isolation. Plasma isolated directly after sampling in K_2_EDTA tubes and plasma isolated within one week from preservative Streck tubes provided high cfDNA yield. We demonstrate that contaminating cellular DNA may be challenging to detect and that quantitative polymerase chain reaction and parallel capillary electrophoresis provide complementary information. In summary, reliable cfDNA analysis requires optimized experimental workflows, where the effects of pre-analytical factors should be considered in study designs and in clinical implementations.

**Supplementary Information:**

The online version contains supplementary material available at 10.1038/s41598-025-03508-4.

## Introduction

The use of liquid biopsies is rapidly intensifying in research and clinical routine. Liquid biopsy-based biomarker analysis has gained substantial attention since several body fluids can be collected with minimal invasiveness, thus enabling repeated sampling and allow for assessment of essentially any type of analyte, including DNA, RNA, proteins and metabolites^1^. Analysis of cell-free DNA (cfDNA) in blood plasma has been demonstrated to be useful in several medical areas, including prenatal testing^2^, cancer^3^, transplantation medicine^4^, vascular disease^5,6^, neurodegenerative disease^7,8^, autoimmune disease^9,10^ and forensic medicine^11,12^. In some areas, such as cancer management, several applications exist, including screening of asymptomatic individuals, diagnosis, prognostication, treatment prediction, monitoring of treatment efficacy as well as early detection of treatment resistance and relapse^13,14^. Cell-free DNA can be assessed by different means to provide clinically relevant information, including total amount of cfDNA, mutations, fragmentation patterns and DNA modifications, such as DNA methylation^15,16^.

The presence of extracellular nucleic acids in blood was first described 1948 by Mandel and Metais^17^. Almost two decades later, Tan et al*.*, reported increased concentrations of cfDNA in patients with developing autoimmune diseases^18^. In cancer treatment, Leon et al*.*, demonstrated 1977 that decreasing cfDNA levels in patients were associated with reduction in tumor volume after radiation therapy^19^, while Stroun et al.^20^ showed that at least a fraction of the total cfDNA originates from tumor cells in cancer. Major challenges when analyzing cfDNA are that the amount is low, generally less than 10 nanogram per milliliter blood plasma in healthy individuals and highly fragmented to a mean size of ~ 167 base-pairs^14^. Longer cfDNA fragments corresponding to di- and tri-nucleosomes are also present at lower concentrations^21,22^. Cell-free DNA is mainly derived from apoptosis and necrosis, but cfDNA may also originate from secretion^23^ or other forms of cell death^24,25^. Cell-free DNA is efficiently cleared from the blood through multiple processes, involving the liver, spleen, kidneys and nucleases^26–29^, and has a half-time between 16 min and ~ 2.5 h^30–32^. Consequently, for most applications, the cfDNA of interest is present at very low concentrations, often with only individual molecules originating from the tissue of interest^33,34^. To enable sensitive and specific cfDNA analysis, methods that allow for detection of individual molecules in a background of sparse and fragmented DNA from numerous cell sources are normally required. During the last decade, numerous approaches have been developed to analyze cfDNA, where most are based on quantitative polymerase chain reaction (qPCR), digital PCR and sequencing technologies^35,36^. In routine blood plasma sampling, standard blood collection tubes are the preferred option, such as K_2_EDTA tubes. These allow for assessment of not only cfDNA but also of other analytes, including proteins and metabolites. However, plasma cannot always be isolated within a given time frame due to logistical reasons, resulting in cellular DNA contamination. Hence, preservative blood collection tubes have been developed to enable plasma isolation even weeks after sampling.

The aim of this study was to establish a pre-analytical workflow with automatic cfDNA extraction, enabling the handling of large numbers of samples. The setup should be compatible with both K_2_EDTA tubes and at least one type of preservative blood collection tube, providing high cfDNA yields without cellular DNA contamination. Hence, we evaluated the performance of standard K_2_EDTA blood collection tubes alongside that of three preservative blood collection tubes with automated magnetic bead-based cfDNA extraction using the QIAsymphony SP system. We analyzed the effect of time between sampling and plasma isolation. To assess the amount of cfDNA, we used fluorometric analysis and qPCR. To determine the degree of contaminating cellular DNA we evaluated several strategies, including qPCR assays that detect long and short DNA fragments, respectively, as well as parallel capillary electrophoresis. Based on our results, we provide insights and recommendations for pre-analytical steps using automatic cfDNA extraction that will help facilitate successful cfDNA analysis.

## Results

### Study and assay design

**Fig. 1:**
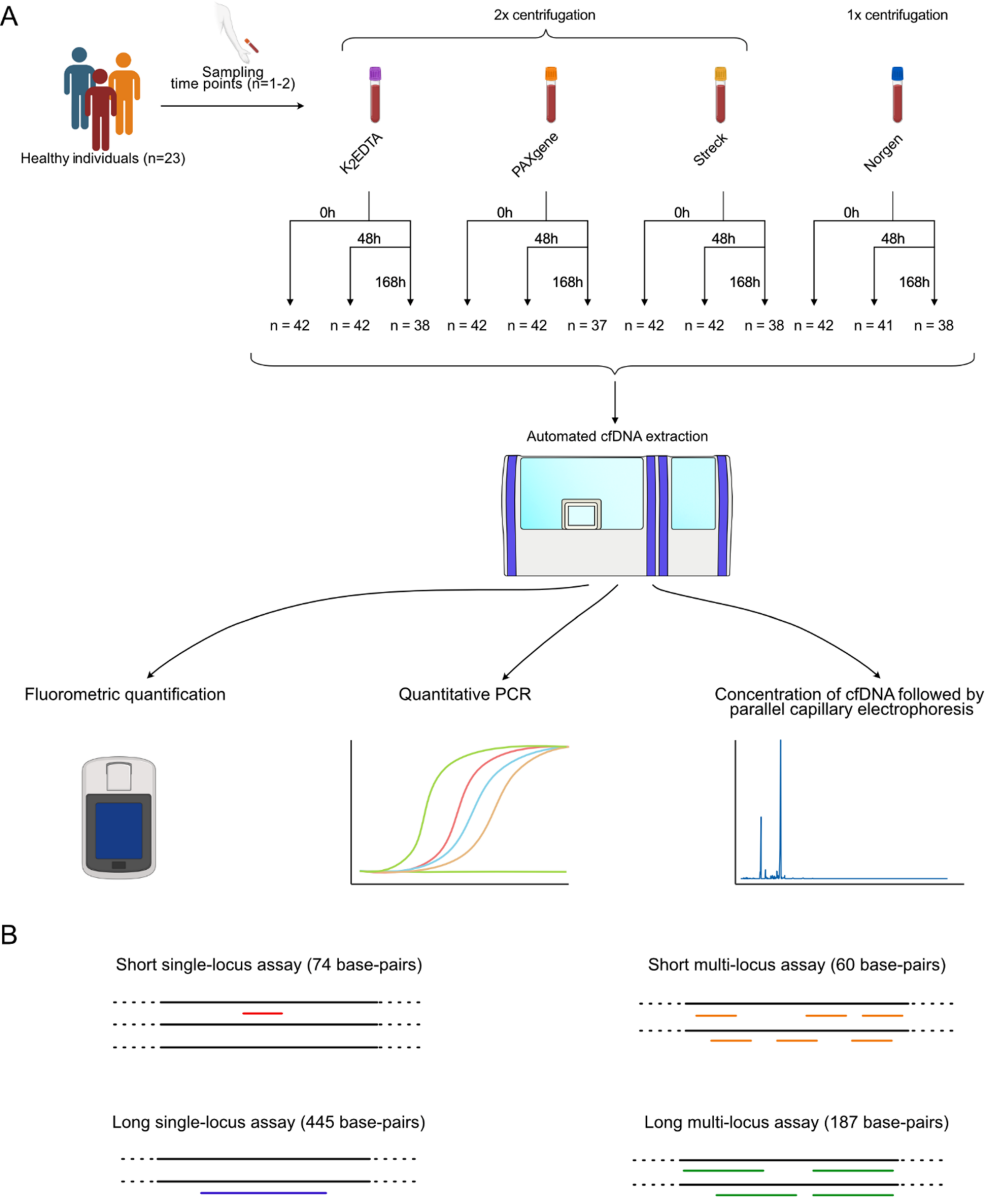
Study overview. (**A**) Blood sampling strategy. Blood samples were collected from 23 healthy individuals, where 20 individuals were sampled twice. For detailed sampling schedule, see Supplementary Fig. S1 and Supplementary Table S1. Blood was drawn into K_2_EDTA, Norgen, PAXgene and Streck tubes and plasma was prepared 0, 48 or 168 h after sampling. Single- or double centrifugation steps were used based on the manufacturers’ recommendations. Plasma from a smaller subset of samples was prepared with deviating number of centrifugations (Supplementary Figs. S1-2). All samples were paired, enabling direct comparisons between pre-analytical conditions. Cell-free DNA was extracted from 1.3–2.0 mL plasma using QIAsymphony SP and magnetic bead-based protocols. The cfDNA concentration was assessed by fluorometric analysis and qPCR. Contamination of cellular DNA in plasma was determined by both qPCR, targeting short and long sequences, and parallel capillary electrophoresis. For the latter, cfDNA was concentrated before analysis. (**B**) Design of qPCR assays. To assess total cfDNA yield, we used qPCR assays targeting short sequences, while qPCR assays targeting long sequences were used to detect cellular DNA. Both single- and multi-locus assays were applied.

To study the effect of pre-analytical parameters on cfDNA yield and purity, we analyzed blood plasma collected from 23 healthy individuals (Fig. 1A). Twenty of these individuals were sampled twice with on average 47.6 days between the two sampling time points (Supplementary Table S1). The rationale for sampling the same individuals twice was that these samples were intended to serve as healthy controls in studies monitoring cancer patients over time. In addition, we also had an interest in assessing cfDNA levels in healthy individuals over time. We collected blood in four different blood tubes, including standard BD Vacutainer PPT Plasma Preparation (K_2_EDTA) tubes and three preservative tube types: cf-DNA/cf-RNA Preservative (Norgen) tubes, PAXgene Blood ccfDNA (PAXgene) tubes and Cell-Free DNA BCTs (Streck) tubes. The additives that preserve blood plasma are osmotic cell stabilizers in Norgen, preventors of apoptosis in PAXgene and chemical crosslinking in Streck^37^. To assess the effect of time between blood sampling and plasma isolation, we centrifuged the plasma after 0 (< 60 min), 48 and 168 h. We also evaluated the effect of single and double centrifugation steps, where plasma prepared from K_2_EDTA, PAXgene and Streck tubes is normally centrifuged twice, while plasma prepared from Norgen tubes is centrifuged only once. All pre-analytical conditions tested were performed on samples derived from the same individuals and time points, enabling paired analyses.

To assess the amount of cfDNA, we used fluorometric analysis and qPCR. For qPCR, we applied two types of short assays, targeting single-locus or multi-locus sequences, respectively (Fig. 1B). The single-locus assay targeted a 74 base-pairs sequence in the *PDGFRA* gene, while the multi-locus assay targeted a 60 base-pairs consensus Alu sequence. To identify contaminating cellular DNA in cfDNA, we also applied qPCR assays targeting longer sequences than the typical cfDNA length of ~ 167 base-pairs. The long single-locus assay targeted a 445 base-pairs sequence in the *FLI1* gene, while the long multi-locus assay targeted a 187 base-pairs consensus Alu sequence. By comparing the ratio between the amount of DNA detected by the long qPCR assay with the amount of DNA detected by the short qPCR assay, the fraction of cellular DNA can be assessed with the assumption that cellular DNA remains, at least partly, unfragmented. This ratio can be calculated for the single-locus and multi-locus assays, respectively. Another method to assess fragment sizes is parallel capillary electrophoresis that provides electropherogram with size distribution of all present DNA molecules in the sample. However, parallel capillary electrophoresis is in comparison with qPCR less sensitive. Hence, the cfDNA was concentrated before analysis.

### The cfDNA yield depends on type of blood collection tube and time between sampling and plasma isolation

**Fig. 2:**
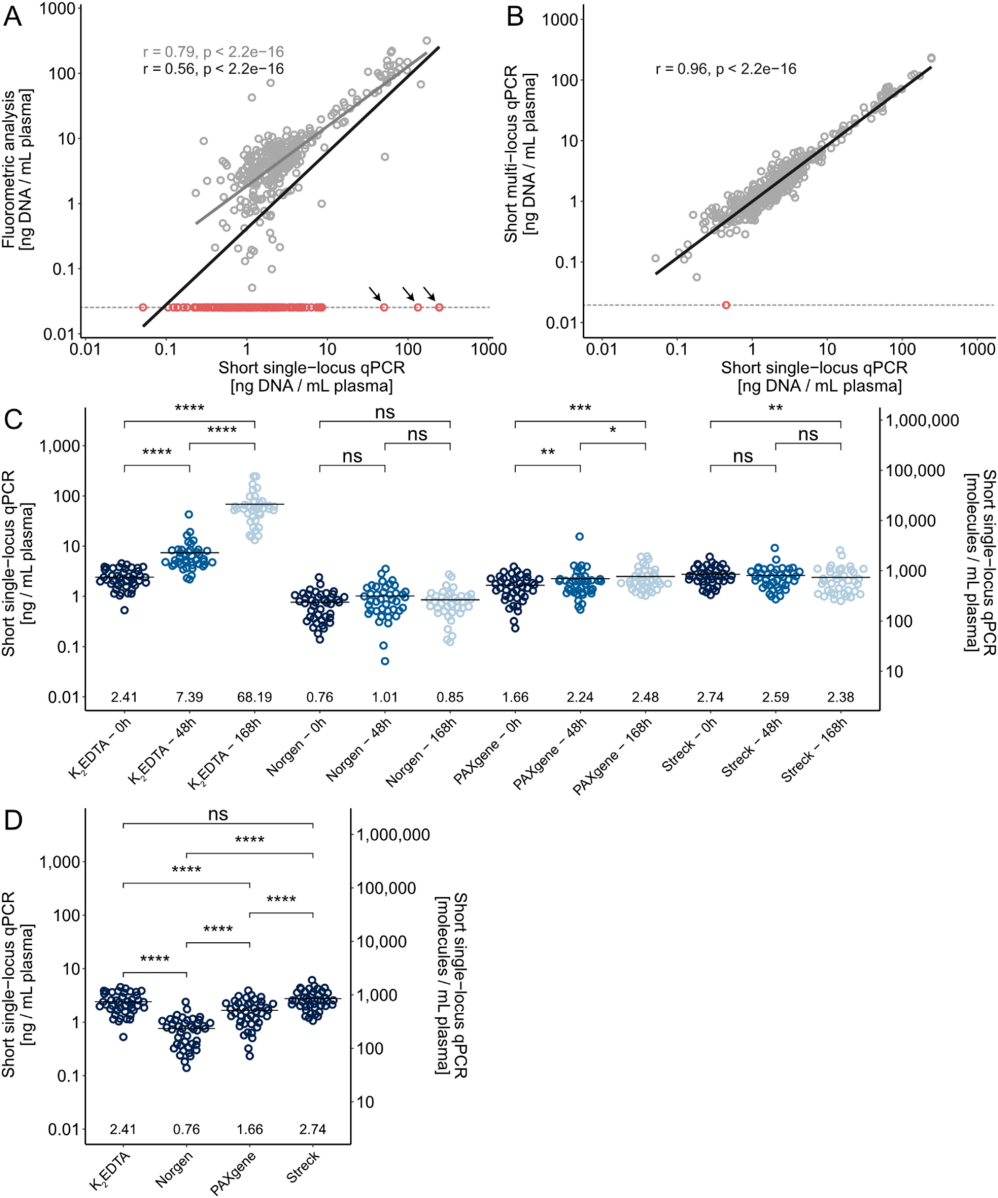
Quantification of cfDNA concentration in blood plasma. (**A**) Comparison of cfDNA concentrations determined by fluorometric analysis and short single-locus qPCR. Linear regression is shown for all data (black line, n = 486) and for data with detectable cfDNA using fluorometric analysis (grey line, n = 343). Pearson correlation coefficients (r) were calculated. Dashed line corresponds to lowest quantified cfDNA concentration divided by two. Samples with no fluorometric readout due to too low cfDNA concentrations are shown in red (n = 143). Three samples marked with arrows were considered technical outliers. (**B**) Concentration of cfDNA quantified using single-locus versus multi-locus qPCR assays. Linear regression is shown (n = 486). Pearson correlation coefficient was calculated. Dashed line corresponds to lowest quantified cfDNA concentration divided by two. One sample marked in red was considered a technical outlier using short multi-locus qPCR. (**C**) Concentration of cfDNA collected in K_2_EDTA, Norgen, PAXgene and Streck tubes with plasma isolation after 0, 48 and 168 h. Plasma from K_2_EDTA, PAXgene and Streck tubes was centrifugated twice, while plasma from Norgen tubes was centrifugated once, according to manufacturers’ instructions. The mean cfDNA concentration is indicated by a bar and below the data points. Wilcoxon signed-ranked test was used, * p ≤ 0.05, ** p ≤ 0.01, *** p ≤ 0.001, **** p ≤ 0.0001 and ns, not significant. (**D**) Concentration of cfDNA collected in K_2_EDTA, Norgen, PAXgene and Streck tubes with plasma isolation at 0 h. Data were rearranged from subfigure C for visualization purposes. Wilcoxon signed-ranked test was used, **** p ≤ 0.0001 and ns, not significant.

In total, we extracted cfDNA from 649 blood plasma samples (Fig. 1A and Supplementary Figs. S1-2). First, we analyzed the cfDNA concentration, following the manufacturers’ recommended number of plasma centrifugation steps, using fluorometric analysis, where 343 of 486 samples generated detectable cfDNA levels. Next, we assessed the cfDNA concentration using qPCR, where cfDNA was detected in all samples using the single-locus assay. We observed a linear correlation between cfDNA concentration assessed by fluorometric analysis versus qPCR, where the variability increased with decreasing cfDNA concentrations (Fig. 2A). Figure 2B shows the correlation between single-locus and multi-locus qPCR assays. Here, we observed a high correlation between the two assays regardless of the cfDNA concentration. For the multi-locus assay, we detected cfDNA in all samples except one.

Figure 2C shows the cfDNA concentrations for the four blood collections tubes using the recommended number of plasma centrifugations and when the time between blood sampling and plasma isolation was 0, 48 or 168 h. Using the single-locus assay, we could also estimate the number of molecules per mL plasma. At 0 h, we detected on average 2.74, 2.41, 1.66 and 0.76 ng cfDNA/mL plasma for Streck, K_2_EDTA, PAXgene and Norgen tubes, respectively (Fig. 2D). For K_2_EDTA tubes, the cfDNA concentrations increased over time to 7.39 and 68.19 ng cfDNA/mL plasma at 48 and 168 h, respectively. For the three preservative blood collection tubes the cfDNA concentrations varied less over time, but we detected a 49.4% increase in cfDNA yield for PAXgene tubes when comparing 0 with 168 h, while a 13.1% decrease in cfDNA yield for Streck tubes when comparing 0 with 168 h. The cfDNA yield in Norgen tubes remained stable over time. Similar data were obtained using the short multi-locus qPCR assay (Supplementary Fig. S3). Next, we compared the effect of one versus two centrifugations in paired samples (Supplementary Fig. S4). For K_2_EDTA, Norgen and PAXgene tubes, the cfDNA concentrations were overall higher for plasma with one centrifugation compared to two centrifugation steps, while no differences were observed for Streck tubes.

**Fig. 3:**
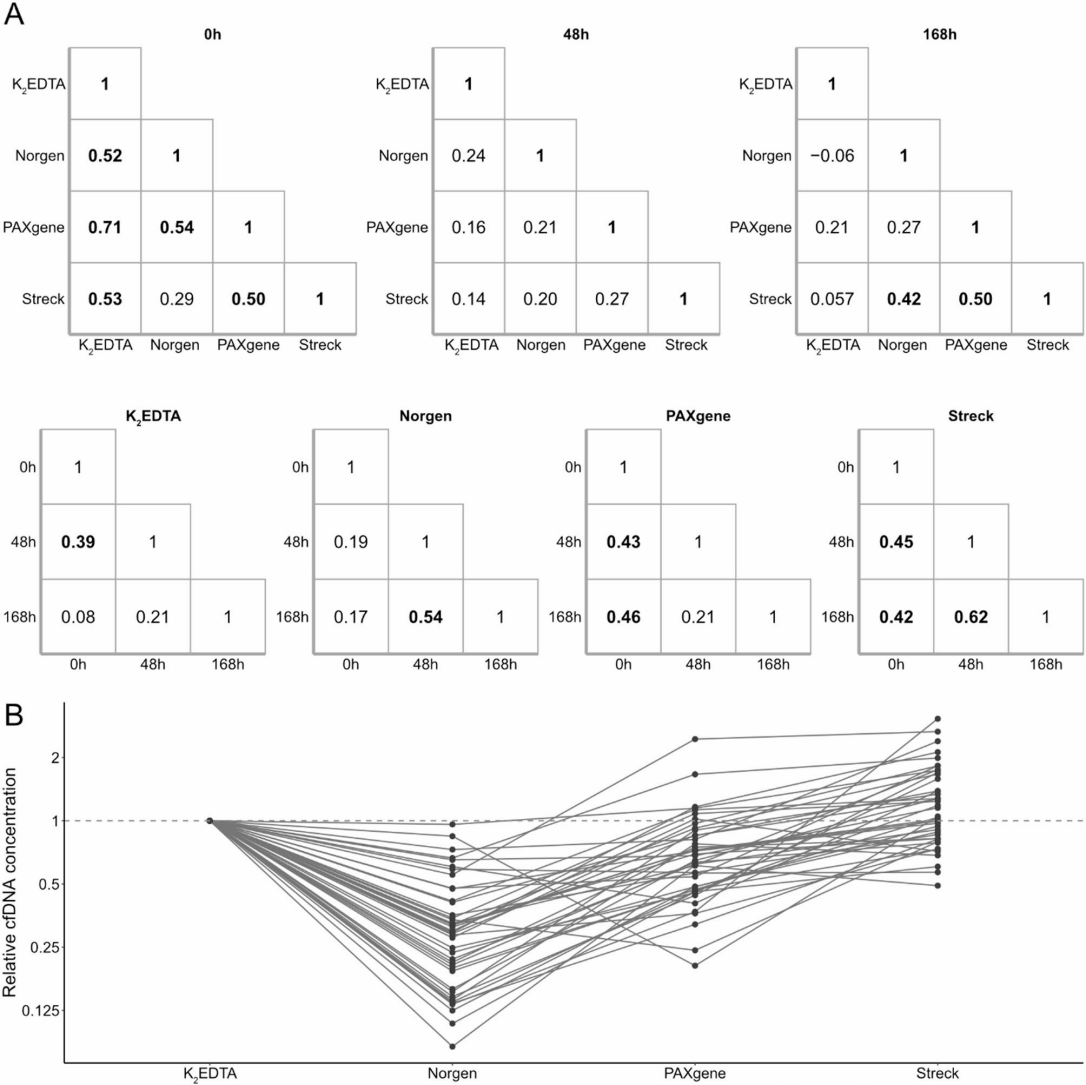
Correlations between cfDNA concentrations comparing blood collection tube types and sampling time points. (**A**) Pair-wise correlations of cfDNA concentrations between tube types as well as time from sampling to plasma isolation. Pearson correlation coefficients were calculated using single-locus qPCR data. Values shown in bold were considered significant, p < 0.05. (**B**) Relative cfDNA concentrations between blood collection tubes at 0 h. If the tubes performed identically to each other the relative cfDNA yield would be equal to one since the blood was sampled from the same individual at the same time point into the four different blood collection tube types. Data are normalized to cfDNA concentrations obtained from K_2_EDTA tubes for visualization purposes, n = 42.

Blood was sampled for all conditions at the same time for each individual. Hence, we could directly compare the cfDNA yield for each sample in relation to blood collection tube and time between sampling and plasma isolation (Fig. 3A). At 0 h, the cfDNA concentrations correlated significantly between all blood collection tubes except between Norgen and Streck tubes. The correlation was highest between K_2_EDTA and PAXgene (r = 0.71), followed by PAXgene and Norgen (r = 0.54) and between K_2_EDTA and Streck (r = 0.53). The correlations generally decreased when the time between sampling and plasma isolation increased. At 168 h, only correlations between Norgen and Streck (r = 0.42) and between PAXgene and Streck (r = 0.50) were observed. Figure 3B visualizes the variability between blood collection tube types at 0 h. For example, the relative cfDNA concentration obtained by Steck compared with K_2_EDTA tubes ranged between 0.49 and 3.1 times, despite that samples were collected from the same individual at a given time point. We also compared the cfDNA concentrations between time points for each blood collection tube (Fig. 3A). As expected, the cfDNA concentrations correlated poorly across the three time points for K_2_EDTA tubes. The three preservative tube types displayed somewhat higher correlations when comparing cfDNA concentrations over time, where Streck tubes displayed the highest correlations. Finally, we also compared whether the cfDNA concentration changed between the two sampling time points for each individual. Supplementary Figure S5 shows that the cfDNA concentrations varied on average 36% from the first to the second time points for the 20 individuals sampled twice. We observed that individuals with comparably high cfDNA concentration at the first time point remained at a higher level also at the second time point and vice versa, indicating that individuals have their own cfDNA concentration baseline. We observed no statistical differences in cfDNA yield based on gender (Mann–Whitney U-test) nor any correlation between cfDNA yield and age (Pearson correlation).

### Cellular DNA contamination is dependent on blood collection tube type, time between sampling and plasma isolation as well as number of centrifugations

**Fig. 4:**
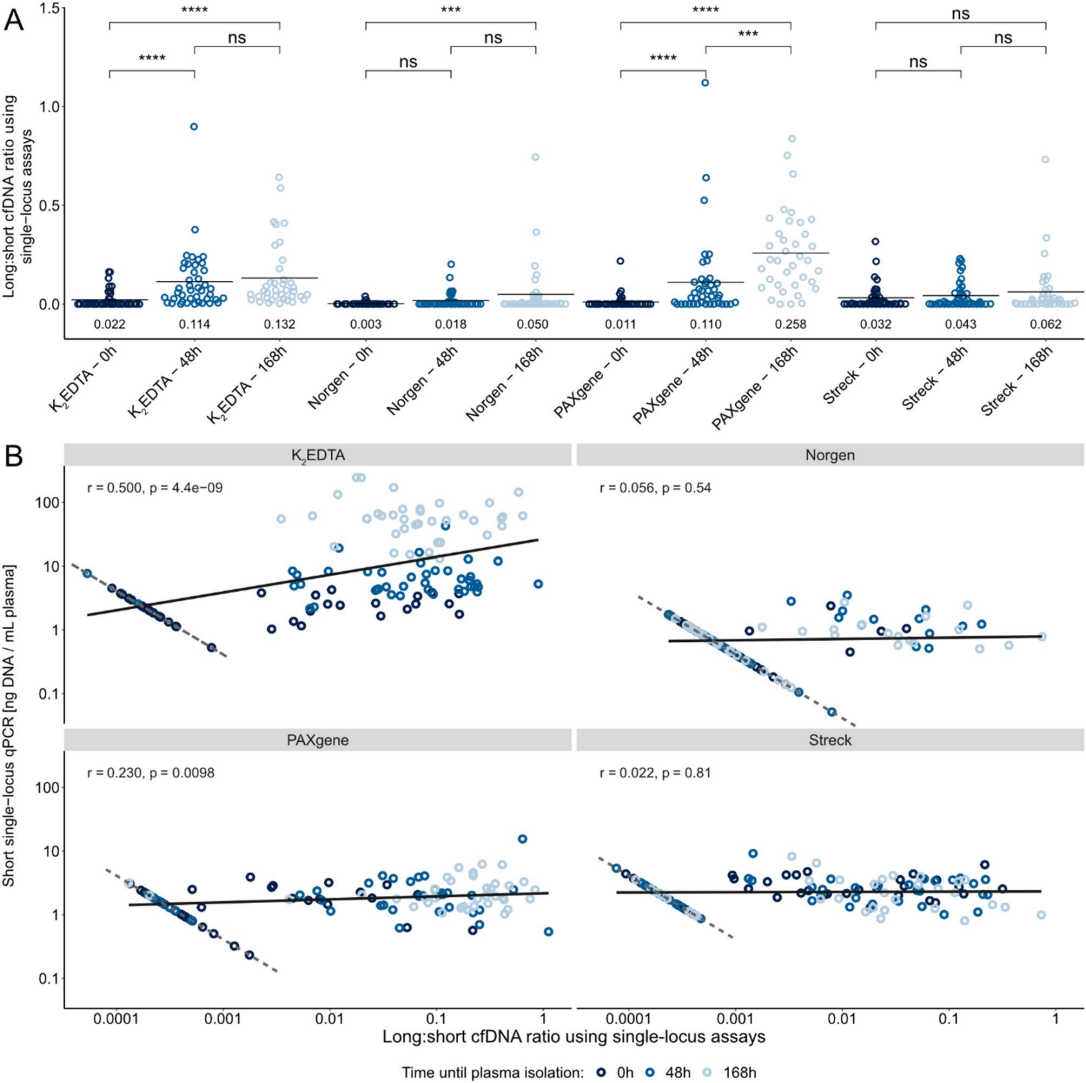
Cellular DNA contamination assessed by qPCR. (**A**) The long:short cfDNA ratio using single-locus qPCR is shown. The mean long:short cfDNA ratio is indicated by a bar and below the data points. Wilcoxon signed-ranked test was used, *** p ≤ 0.001, **** p ≤ 0.0001 and ns, not significant. (**B**) Correlations between long:short cfDNA ratio and cfDNA concentration. The linear fit is shown by solid line. Samples with no cfDNA detected by the long qPCR assay are highlighted by the dashed line. This ratio decreased with increasing cfDNA concentrations since missing data generated by the long qPCR assay were replaced by a constant value, while the value for the short qPCR assay increased with the cfDNA concentration. Pearson correlation coefficients (r) were calculated using all data.

Our data clearly show that the cfDNA concentrations in K_2_EDTA plasma increased with time, likely due to contaminating cellular DNA from dying leukocytes. To assess the degree of cellular contamination, we assessed the ratio of cfDNA levels quantified by long and short single-locus qPCR assay, respectively (long:short cfDNA ratio, Fig. 4). The long:short cfDNA ratio in average increased from 0.02 at 0 h to 0.11 at 48 h and 0.13 at 168 h for K_2_EDTA plasma. For the PAXgene tubes, the long:short cfDNA ratios were even higher than for the K_2_EDTA tubes at 168 h. For Norgen tubes, we observed a small increase in long:short cfDNA ratio, while no cellular contamination was observed for Streck tubes. We also calculated the long:short cfDNA ratio using multi-locus assays (Supplementary Fig. S6). Here, the same trends were observed, but the increase was lower for PAXgene tubes than for K_2_EDTA tubes when comparing data for 0 with 168 h. In addition, we also observed a higher long:short cfDNA ratio for Norgen tubes. Figure 4B shows the association between long:short cfDNA ratio and cfDNA concentration based on the single-locus qPCR assays. Data show a positive correlation for K_2_EDTA tubes (r = 0.50) and PAXgene tubes (r = 0.23), confirming that both tube types are contaminated with cellular DNA when plasma samples were not isolated directly after sampling. Interestingly, we observed both high and low long:short cfDNA ratios for K_2_EDTA tubes with elevated cfDNA concentrations, indicating that qPCR may not always detect cellular contamination.

**Fig. 5:**
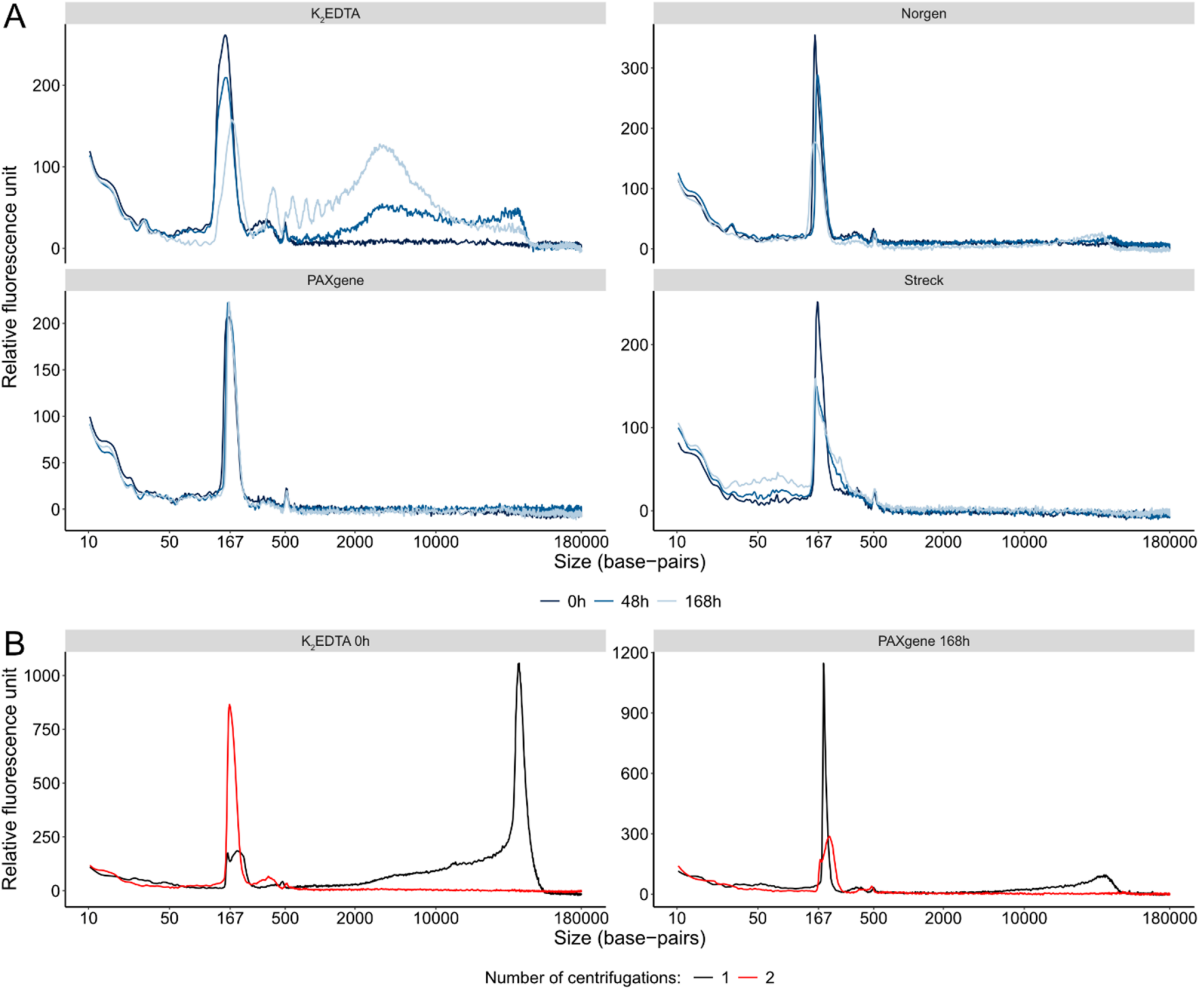
Cellular DNA contamination assessed by parallel capillary electrophoresis. (**A**) Electropherograms for K_2_EDTA, Norgen, PAXgene and Streck tubes with plasma isolation performed after 0, 48 and 168 h. Data are shown for individual 4. Additional data are shown in Supplementary Fig. S7. (**B**) Electropherograms representative for single and double centrifugated plasma samples. Data are shown for individual 18. Additional data are shown in Supplementary Fig. S9.

We analyzed a subset of samples with parallel capillary electrophoresis to provide more comprehensive cfDNA fragment data (Fig. 5 and Supplementary Fig. S7). For the K_2_EDTA tubes at 48 and 168 h, we detected longer cellular DNA fragments compared with 0 h. At 168 h, we also observed peaks with ~ 167 base-pairs intervals. For the preservative blood collection tube samples, we observed the characteristic cfDNA peak around 167 base-pairs together with short, < 50 base-pairs, fragments and smaller traces of longer fragments. For the PAXgene and Streck tubes we often observed a somewhat wider peak around 167 base-pairs for the 48 and 168 h samples compared to 0 h samples.

Finally, we assessed the effect of single versus double centrifugation in relation to cellular contamination. Supplementary Figure S8 shows that the degree of cellular DNA contamination, detected by long versus short qPCR single-locus assays, tended to be higher in single versus double centrifugated plasma from all tube types except for Streck tubes, but the variation between samples was high. Figure 5B and Supplementary Fig. S9 clearly show that an extra centrifugation step efficiently removes major fractions of contaminating cellular DNA with large sizes for a subset of samples.

## Discussion

Cell-free DNA analysis is applied in an increasing number of applications. Numerous experimental tools have been developed with the technical sensitivity to detect individual cfDNA molecules as means to determine disease and health conditions. There are many pre-analytical reports evaluating different steps and parameters, including choice of blood collection tube types and time between sampling and plasma isolation^38–43^. Table 1 summarizes 27 pre-analytical studies, focusing on automated extraction protocols and methods to assess cfDNA yield and quality. Few studies use the same analytical approaches or evaluate parameters the same way, such as a given combination of blood collection tubes, time between sampling and plasma isolation and extraction method. Thus, conclusions and recommendations are therefore somewhat contradictory. In comparison to these studies, our study provides new insights into the use of an automatic cfDNA extraction approach, enabling clinical studies consisting of large number of blood samples, where we have evaluated standard K_2_EDTA blood collection tubes and three preservative tube types. Our study is also performed on a relatively large number of paired samples, providing reliable data on evaluated pre-analytical parameters. It also provides novel information about cfDNA yield and quality by using an extensive number of analytical methods.

**Table 1:** Studies of pre-analytical parameters.

Study	Tube type	Multiple time points	Automated extraction	Fluorometric quantification	Single-locus qPCR	Multi-locus qPCR	Electrophoresis	Other assessments
Alidousty et al.^52^	S,P,R	Yes	Yes	No	Other	–/–	No	No
Barták et al.^53^	E,S	Yes	Yes	Yes	–/–	–/–	No	Yes
Deleu et al.^54^	E,Ci	No	Yes	No	–/–	–/–	Yes	Yes
Lampignano et al.^55^	S,P	No	Yes	Yes	S/L	S/L	Yes	Yes
Lehle et al.^56^	S	No	Yes	Yes	–/–	S/L	Yes	Yes
Maass et al.^37^	E,S,P,N	Yes	No	Yes	–/–	–/–	Yes	Yes
Markus et al.^57^	E,S	Yes	Yes	Yes	–/–	–/–	Yes	Yes
Mehrotra et al.^58^	E,S	Yes	No	Yes	–/–	S/L	Yes	Yes
Nikolaev et al.^59^	E,S,P,R	Yes	No	Yes	S/L	–/–	Yes	No
Parackal et al.^60^	E,S,R	Yes	No	Yes	–/–	–/–	Yes	No
Pedini et al.^61^	R	N/A	Yes	Yes	–/–	–/–	Yes	Yes
Pérez-Barrios et al.^62^	E	No	Yes	Yes	–/–	–/–	Yes	Yes
Polatoglou et al*.*^63^	E	No	Yes	Yes	–/–	–/–	Yes	No
Risberg et al.^64^	E,S	Yes	No	No	–/–	–/–	No	Yes
Samoila et al.^65^	E,S,P	Yes	Yes	No	–/–	–/–	Yes	Yes
Schmidt et al.^66^	E,P	Yes	Yes	No	S/L	–/–	No	Yes
Stasik et al.^67^	E,S,P	Yes	Yes	No	S/–	–/–	No	Yes
Streleckiene et al.^68^	E	No	No	Yes	–/–	–/–	Yes	No
Streubel et al.^69^	E,P	No	Yes	Yes	S/–	–/–	Yes	Yes
Terp et al.^48^	E	No	Yes	No	–/–	–/–	Yes	Yes
van Dessel et al.^70^	E,S,Cs	Yes	No	Yes	–/–	–/–	No	Yes
van Dessel et al.^46^	E,Cs	Yes	Yes	Yes	S/–	–/–	No	Yes
van Ginkel et al.^71^	E,S,Cs,Ci,H	Yes	Yes	Yes	–/–	–/–	No	Yes
Ward Gahlawat et al.^47^	E,S,P,N,R	Yes	No	Yes	–/–	–/–	Yes	Yes
Warton et al.^72^	E,S,P	Yes	No	No	S/–	S/L	Yes	No
Wolf et al.^73^	E,S	Yes	Yes	Yes	–/–	S/–	Yes	Yes
Zhao et al.^74^	E,S,R	Yes	No	No	–/–	S/–	No	Yes

Tube type: S, Streck; P, PAXgene; R, Roche Cell-Free DNA Collection Tube; E, EDTA; Ci, Citrate; N, Norgen; Cs, CellSave preservative tubes; H, Heparin. Multiple time points: different times between sample collection and plasma isolation were tested. Single-locus qPCR assessing short (S, < 150 bp) and long (L, > 200 bp) target sequences. Other: assay of unknown target(s) and size. Multi-locus qPCR assessing short (S, < 150 bp) and long (L, > 200 bp) target sequences.

The extraction efficiencies of different purification methods are known to depend on blood collection tube type^44^. Choice of extraction protocol may not only affect yield but also enrich for specific cfDNA fragment sizes^45^. Here, we used the automated QIAsymphony SP system that has been reported to perform better than the Maxwell RSC instrument for EDTA and CellSave blood collection tubes^46^. Interestingly, we observed an overall large variability between all samples collected at the same time point for each individual, regardless of blood collection tube type, time between sampling and plasma isolation and number of centrifugations steps. The lower yield for plasma collected in Norgen and PAXgene tubes may at least partially be explained by the fact that the plasma composition with additives for osmotic cell stabilization in Norgen and additives that prevent apoptosis in PAXgene may be less compatible with the applied magnetic bead-based extraction. In contrast, Norgen and PAXgene tubes have been shown to outperform Streck tubes using silicon membrane-based spin columns for cfDNA extraction that is based on other extraction chemistries^47^. Another factor is that the additives in question may affect plasma volume. For the Norgen plasma, we also performed a somewhat higher centrifugal force than recommended since we prioritized minimizing the variation in timing between sampling and plasma isolation between tube types. Hence, some of the detected variations in our data may be attributed to suboptimal handling of plasma isolation. It should be noted that manual cfDNA extraction protocols, such as QIAamp Circulating Nucleic Acid Kit, have been reported to provide higher cfDNA yields than automated cfDNA extraction protocols^48^.

Quantitative PCR outperformed fluorometric analysis and single-locus qPCR assays provided similar results as multi-locus qPCR assays. While data from single-locus assays can be converted to molecule and cell numbers with ease, results from multi-locus assays may be preferred in cfDNA applications with expected aneuploidy, such as in cancer management. However, the number and nature of Alu repeats may also vary between individuals. As expected, K_2_EDTA plasma generated increased cfDNA concentrations when plasma was isolated first two days after sampling. However, we also noted changes for PAXgene and Streck tubes over time, while plasma from Norgen tubes remained stable. Interestingly, our data show that detection and quantification of contaminating cellular DNA is not trivial. For qPCR data, we preferred the use of the long single-locus assay since it targets a sequence that is ~ 278 base-pairs longer than the most prevalent cfDNA fragment, while the long multi-locus assay targets a sequence that is only ~ 20 base-pairs longer. Other, longer multi-locus sequences can potentially be applied that more easily identify long cellular DNA fragments, such as LINE1^49^. Our data show that the pool of both short and long cfDNA fragments continuously increases over time in K_2_EDTA tubes, were longer DNA fragments sometimes, but not always, could be observed. The variations in the long:short cfDNA ratio between individual K_2_EDTA tubes were high for plasma isolated at both 48 and 168 h after sampling despite that the cfDNA concentrations increased several times over time. Parallel capillary electrophoresis provides more detailed assessment of different DNA fragment sizes. However, this method lacks sensitivity to detect low abundant fragments even after considerable concentration of cfDNA. One reason we are not able to detect all contaminating cellular DNA is due to ongoing nuclease activity, evident by the characteristic oligonucleosomal fragments observed in several electropherograms (Fig. 5, Supplementary Fig. S7 and Supplementary Fig. S9), particularly in K_2_EDTA tubes, resulting in DNA fragments that are indistinguishable from cfDNA in size.

The performance of the preservative tubes varied. Overall, Norgen and Streck tubes maintained similar cfDNA concentrations over time with only a minor decrease in cfDNA concentrations for Streck tubes at 168 h detected. We speculate that this effect may be a result of the chemical crosslinking strategy used in Streck tubes. For PAXgene tubes we observed a systematic, small but significant, leakage of cellular DNA into the plasma over time but the underlying reason is not clear. The long:short cfDNA ratio indicates that the cellular DNA contamination may be similar to that in the K_2_EDTA tubes. However, the overall cfDNA concentration in PAXgene tubes only increased 49%, while the increase was 28 times when comparing plasma isolated in K_2_EDTA tubes directly or after 168 h. Furthermore, the increases in long:short cfDNA ratios using the multi-locus assays were lower compared to the single-locus assays for PAXgene tubes in relation to K_2_EDTA tubes, indicating that the contaminating cellular DNA fragments were longer in PAXgene tubes. A similar trend was observed for Norgen tubes. This is most likely an effect of the specific additives that are used to suppress cellular contamination in both tubes. The parallel capillary electrophoresis analysis showed no clear cellular DNA contamination in PAXgene plasma, but the cfDNA peak was somewhat wider in several PAXgene plasma samples at both 48 and 168 h. It should however be noted that the parallel capillary electrophoresis required that the cfDNA samples had been concentrated prior to analysis. Even then this method displayed comparably low sensitivity to detect low concentrations of specific DNA fragments, such as cellular DNA contamination. In conclusion, qPCR and parallel capillary electrophoresis can only partly detect cellular DNA contamination.

The choice of blood collection tube, time between sampling and plasma isolation and number of centrifugation steps all affect both the cfDNA yield and purity. Most applications depend on both parameters. For example, circulating tumor-DNA in cancer patients is generally reported as variant allele frequencies or as molecules per mL plasma. Confounding factors when assessing variant allele frequencies depend mainly on cfDNA purity and on changes of cfDNA release from non-tumor cells. For data reported as molecules per mL plasma, unwanted variability mainly depends on variations in cfDNA extraction yield^50^. Hence, it is fundamental to report all pre-analytical parameters as they potentially may influence data interpretation. The variability between different pre-analytical strategies delays the implementation of cfDNA analysis in routine applications. One way to overcome this obstacle is to standardize the pre-analytical steps. However, implementing any type of standardization too early may limit the potential use of cfDNA analysis for some applications since methods to collect plasma, extract cfDNA and analyze cfDNA are still in the development phase.

In conclusion, our data show that automated cfDNA extraction using the QIAsymphony SP system provides cfDNA of high yield and quality from plasma that is directly isolated from K_2_EDTA tubes and from plasma that is isolated within a week using preservative Streck tubes. The concentration of cfDNA is preferably determined with qPCR rather than fluorometric analysis and cellular DNA contamination is best assessed using a combination of short and long qPCR assays and parallel capillary electrophoresis.

## Material and methods

### Ethical approval

This study was performed in accordance with the Declaration of Helsinki and approved by the Regional Ethical Review Board in Gothenburg (Dnr: 054–15, date of approval 2015–05-18). Informed consent was obtained from all participants.

### Blood sampling and cell-free DNA extraction

Peripheral blood was collected from 23 healthy individuals in four tube types: K_2_EDTA (BD Vacutainer PPT Plasma Preparation Tubes, BD, #362799), Norgen (cf-DNA/cf-RNA Preservative Tubes, Norgen Biotek, #63950), PAXgene (PAXgene Blood ccfDNA Tubes, PreAnalytiX, #768115) and Streck (Cell-Free DNA BCTs, Streck, #218997) at Sahlgrenska University Hospital Gothenburg, Sweden. Blood collection tubes were kept at room temperature 0 (< 60 min), 48 or 168 h before plasma isolation.

All K_2_EDTA, PAXgene, and Streck samples were centrifuged at 2000 *g* for 10 min at room temperature, while Norgen samples were centrifuged at 2000 *g* for 20 min at room temperature. A subset of samples was centrifuged a second time at 16,000 g for 15 min at 4 °C. All plasma samples were stored at − 80 °C between 83 and 345 days with an average of 209 days until further processing. DNA extraction was performed using a QIAsymphony SP (Qiagen). For plasma isolated from K_2_EDTA, Norgen and Streck tubes, the QIAsymphony DSP Circulating DNA Kit (Qiagen, #937556) according to the circDNA_2000_DSP_V1 protocol was used, while the PAXgene Blood ccfDNA Tube plasma was extracted using the QIAsymphony PAXgene Blood ccfDNA Kit (Qiagen, #768536) according to the PAXcircDNA_STA_2400_V1 protocol. Cell-free DNA was eluted in 60 µL elution buffer.

Cell-free DNA concentration was fluorometrically quantified in 2 µL cfDNA using a Varioskan LUX plate reader (Thermo Fisher Scientific) and the Quant-iT dsDNA high sensitivity (HS) Assay Kit (Thermo Fisher Scientific, #Q33120) or by using Qubit 3.0 Fluorometer (Thermo Fisher Scientific) and Qubit 1X dsDNA High Sensitivity (HS) Assay Kit (Thermo Fisher Scientific, #Q33230). Missing data were replaced by the lowest quantified cfDNA concentration divided by two.

### Quantitative PCR

Quantitative PCR was performed in 10 µL reactions for single-locus (*PDGFRA* and *FLI1*) assays using 1× TATAA SYBR GrandMaster Mix Low Rox (TATAA Biocenter, #TA01-1875LR), 400 nM of each primer (*PDGFRA* forward primer: 5′-GAAGATCTGTGACTTTGGCCTG-3′; *PDGFRA* reverse primer: 5′-GACGTACACTGCCTTTCGAC-3′; *FLI1* forward primer: 5′-TGAGGCTGAATTATCCACAATGGCTGG-3′; *FLI1* reverse primer: 5′-GGGTGTGCCTGCTATGAGAA-3′, desalted, Sigma-Aldrich) and 1 µL cfDNA. For multi-locus assays (Alu-60 and Alu-187), 10 µL reactions contained 1× TATAA Probe GrandMaster Mix (TATAA Biocenter, #TA02-1875), 400 nM of each primer and 200 nM probe (TATAA Biocenter, #QA-01–0939) and 1 µL cfDNA. The four qPCR assays were analyzed in parallel for each sample using a CFX384 Touch real-time PCR detection system (Bio-Rad) with the following temperature profile: 95 °C for 3 min preincubation, followed by 45 cycles of amplification (95 °C for 5 s and 60 °C for 30 s). A melting curve, ranging from 60 to 95 °C (5 s per 0.5 °C increment) was performed to confirm correct PCR product formation for the SYBR Green-based assays. Cycle of quantification values were determined with regression using Bio-Rad CFX Maestro 2.0 (version 5.0.021.0616, Bio-Rad). Assay performance was assessed using standard curve of Human Genomic DNA (Roche, #11,691,112,001), ranging from 64 ng to 0.24 pg with fourfold dilution steps (n = 4). The following PCR efficiencies were obtained: *PDGFRA*: 97.8% (94.7–101.0%), *FLI1*: 90.5% (88.9–92.1%), Alu-60: 101.8% (100.4–103.3%) and Alu-187: 103.2% (100.2–106.1%). The 95% confidence intervals of PCR efficiencies are shown within parentheses. To convert DNA concentrations to molecule numbers for the short single-locus assay we assumed 310 haploid genome equivalents per ng DNA^51^. Missing data were replaced by the lowest cfDNA concentration divided by two. Cellular contamination was assessed as the ratio between *FLI1* and *PDGFRA* as well as the ratio between Alu-187 and Alu-60. See Supplementary Table S2 for experimental data.

### Parallel capillary electrophoresis

Extracted cfDNA was concentrated using Vivacon 500, 30,000 MWCO Hydrosart ultrafiltration spin columns (Sartorius. #VN01H22) to 8 μL. Parallel capillary electrophoresis was performed with a Fragment Analyzer (Agilent Technologies) and the HS Large Fragment 50 kb Kit (Agilent Technologies, #DNF-464-0500), according to the manufacturer’s instructions.

## Electronic supplementary material

Below is the link to the electronic supplementary material.

Supplementary Material 1

Supplementary Material 2

Supplementary Material 3

## Data Availability

All data generated or analysed during this study are included in this published article and its supplementary information files.
