# Supplementary material for: Evaluation of automatic cell free DNA extraction metrics using different blood collection tubes
Source: Sci Rep. 2025 Jun 3;15:19364. doi: 10.1038/s41598-025-03508-4 (PMC12134210; doi:10.1038/s41598-025-03508-4)
Supplement: Supplementary file 1 — Supplementary Material 1 [file 41598_2025_3508_MOESM1_ESM.pdf]

Evaluation of automatic cell free DNA extraction metrics using different blood collection tubes

Daniel Andersson, Helena Kristiansson, Manuel Luna Santamaría, Huma Zafar,

Ivan Mijakovic, Åsa Torinsson Naluai, Anders Ståhlberg

Supplementary Figure 1

Supplementary Figure 2

Supplementary Figure 3

Supplementary Figure 4

Supplementary Figure 5

Supplementary Figure 6

Supplementary Figure 7

Supplementary Figure 8

Supplementary Figure 9

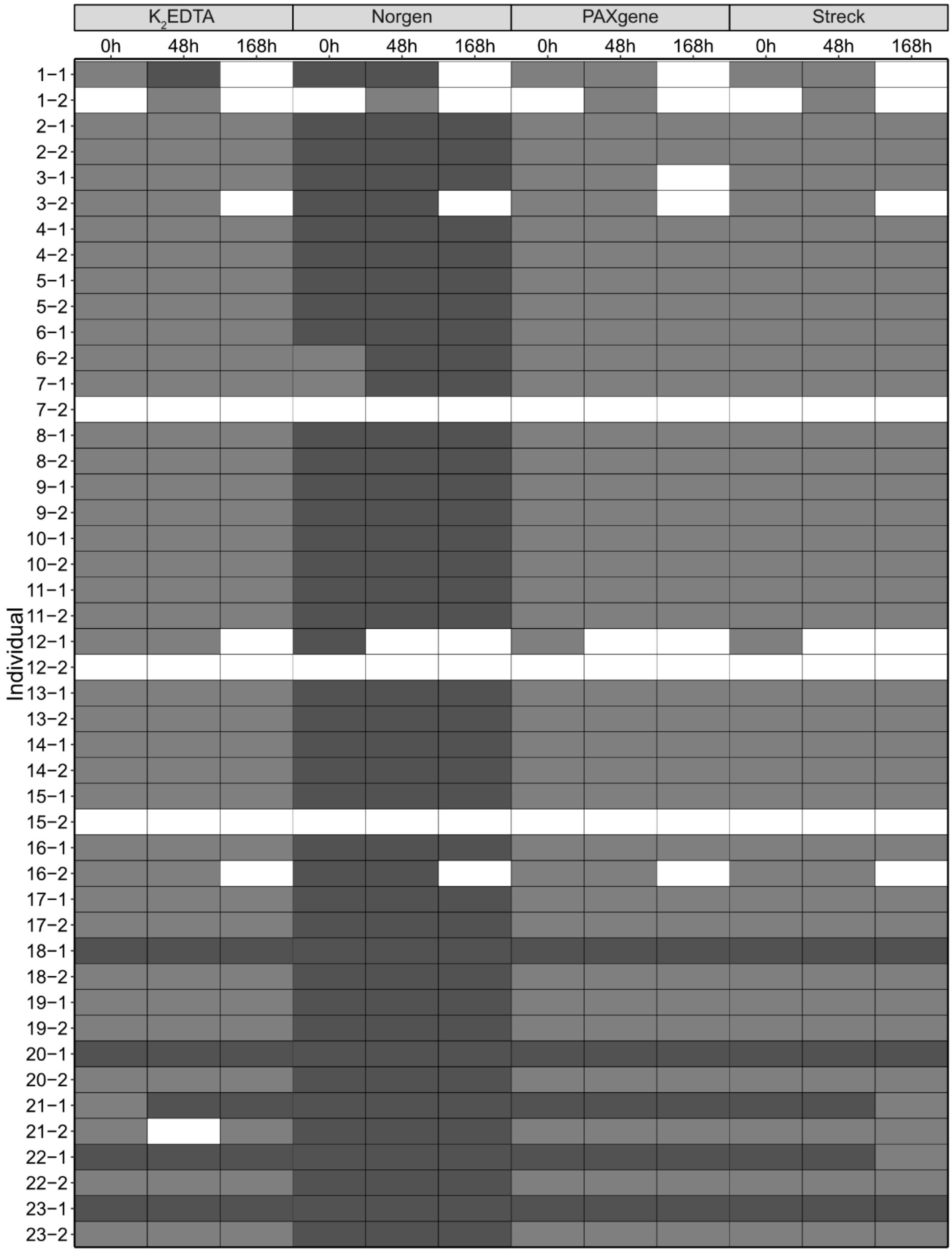

Supplementary Figure S1. Overview of collected samples. Blood samples were collected from 23 healthy individuals, where 20 individuals were sampled twice. Dark grey boxes indicate that samples were collected with both one and two centrifugation steps, while light gray boxes indicate that only one sample was collected with the recommended number of centrifugations. White boxes indicate that no sample was collected.

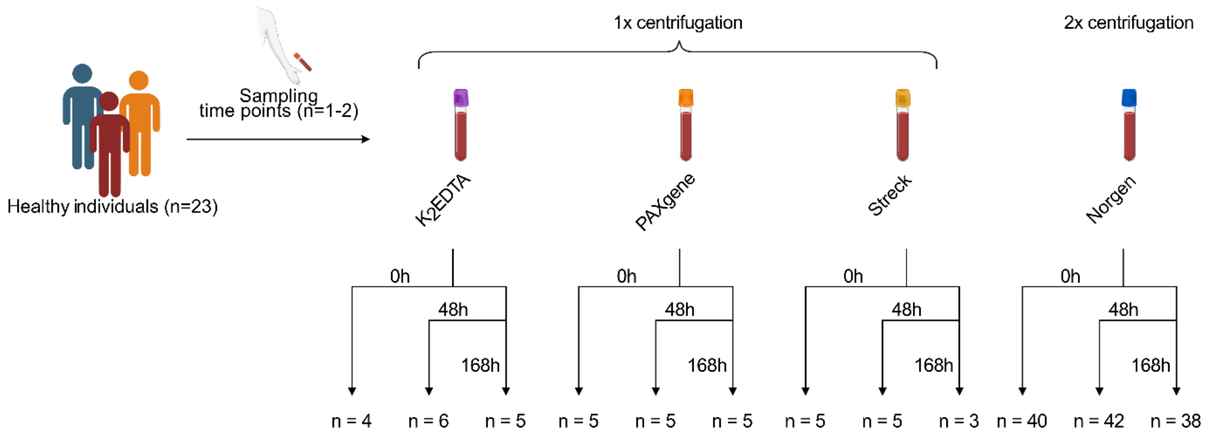

Supplementary Figure S2. Plasma samples with alternative number of centrifugation steps. Blood samples were collected from 23 healthy individuals, where 20 individuals were sampled twice. Blood was drawn into K_2EDTA, Norgen, PAXgene and Streck blood collection tubes and plasma was isolated 0, 48 or 168 hours after sampling using either single or double centrifugation based on the manufacturers' recommendations (outlined in Fig. 1A). Here, plasma was prepared with another number of centrifugations for a subset of samples, *i.e.*, K_2EDTA, PAXgene and Streck plasma samples were centrifuged once, whereas Norgen plasma samples were centrifuged twice.

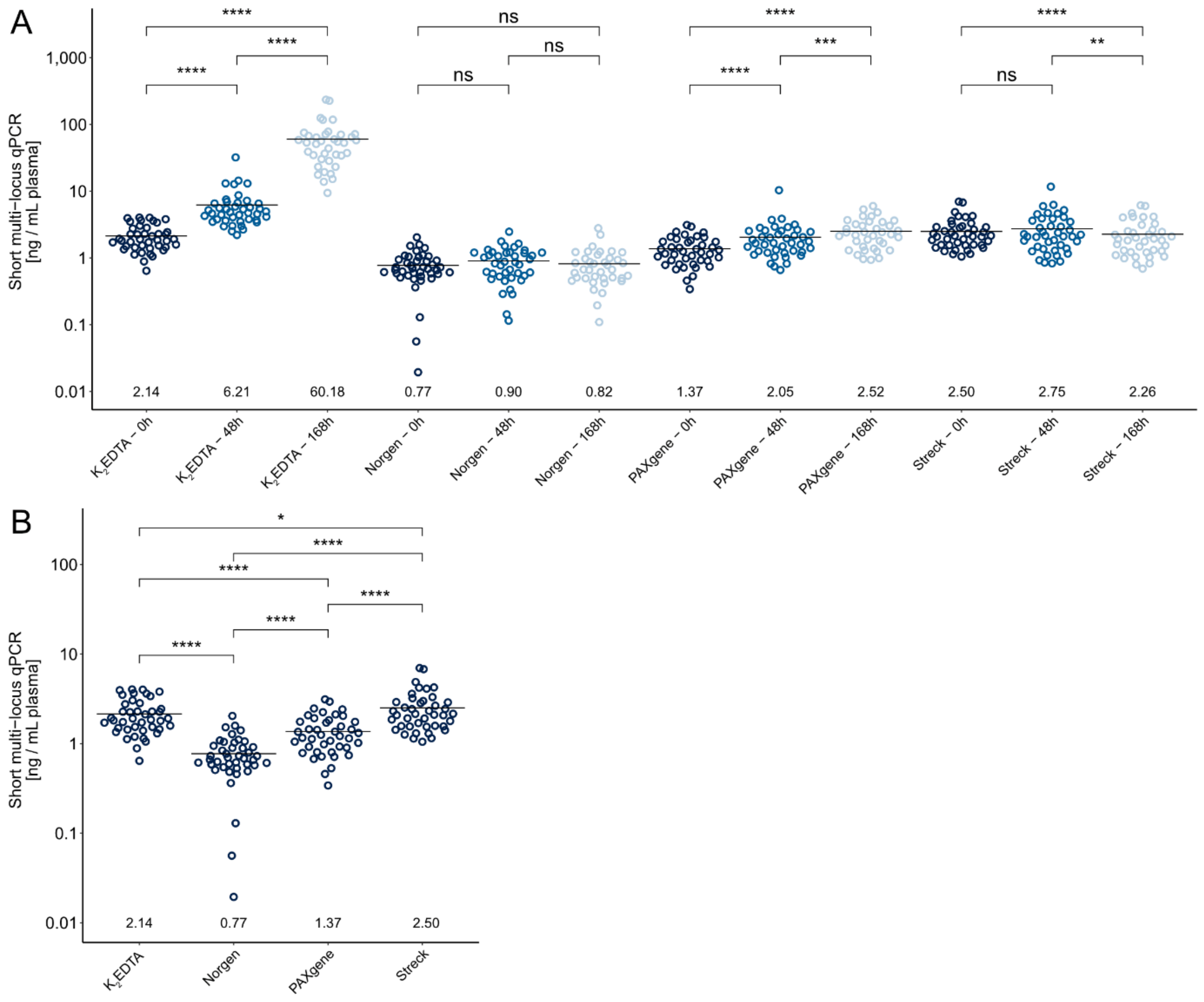

Supplementary Figure S3. Quantification of cfDNA concentration in blood plasma using multi-locus qPCR. (A) Concentration of cfDNA in plasma isolated from K_2EDTA, Norgen, PAXgene and Streck tubes 0, 48 and 168 hours after sampling. K_2EDTA, PAXgene and Streck tubes were centrifuged twice, while Norgen tubes were centrifuged once. The mean cfDNA concentration is indicated by a bar and below the data points. Wilcoxon signed-ranked test was used, ** $p \leq 0.01$, *** $p \leq 0.001$, **** $p \leq 0.0001$ and ns, not significant. **(B)** Comparison of cfDNA concentrations in plasma from different blood collection tubes at 0 hours. Data are rearranged from subfigure A for visualization purposes. Wilcoxon signed-ranked test was used, * $p \leq 0.05$ and **** $p \leq 0.0001$.

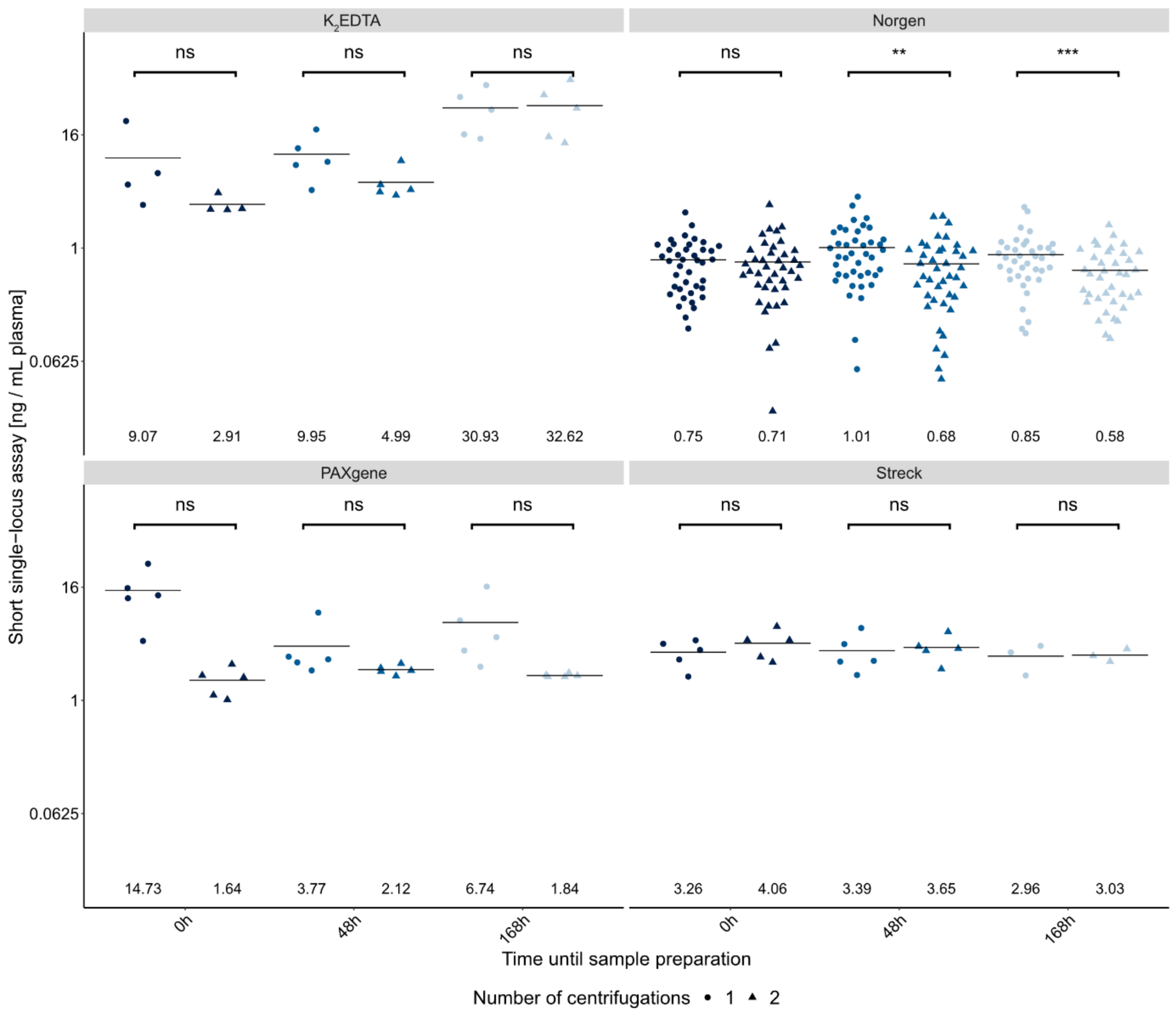

Supplementary Figure S4. Cell-free DNA concentrations in single and double centrifugated plasma. Concentration of cfDNA collected in K_2EDTA, Norgen, PAXgene and Streck tubes with plasma isolation at 0, 48 and 168 hours using either single or double centrifugation steps. Only data from paired samples shown. The mean cfDNA concentration is indicated by a bar and below the data points. Wilcoxon signed-ranked test was used, ** $p \leq 0.01$, *** $p \leq 0.001$ and ns, not significant. Note that the number of samples for K_2EDTA, PAXgene and Streck tubes are too few to reach statistical significance using non-parametric tests.

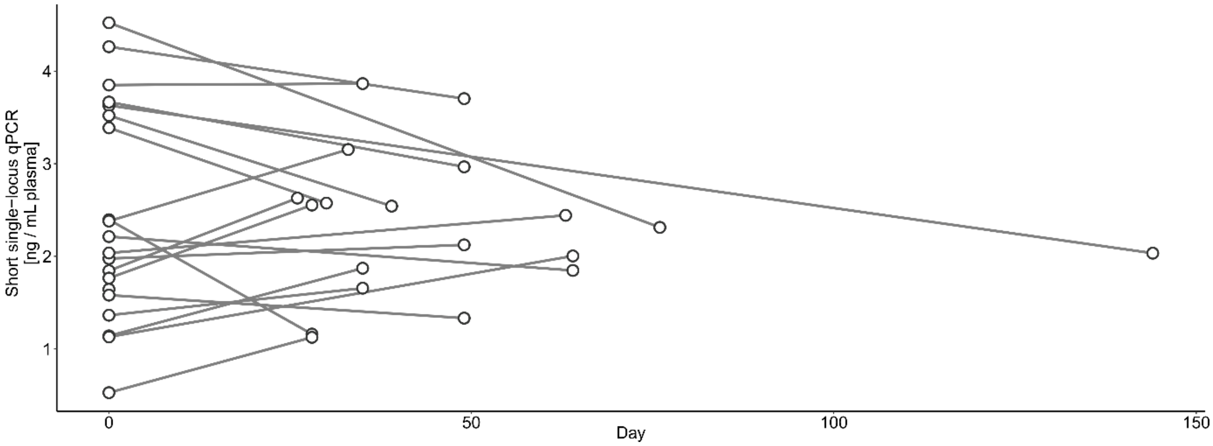

Supplementary Figure S5. Cell-free DNA concentrations between different sampling time points for each individual. Values for single-locus qPCR data on K_2EDTA 0h tubes are shown, n = 20.

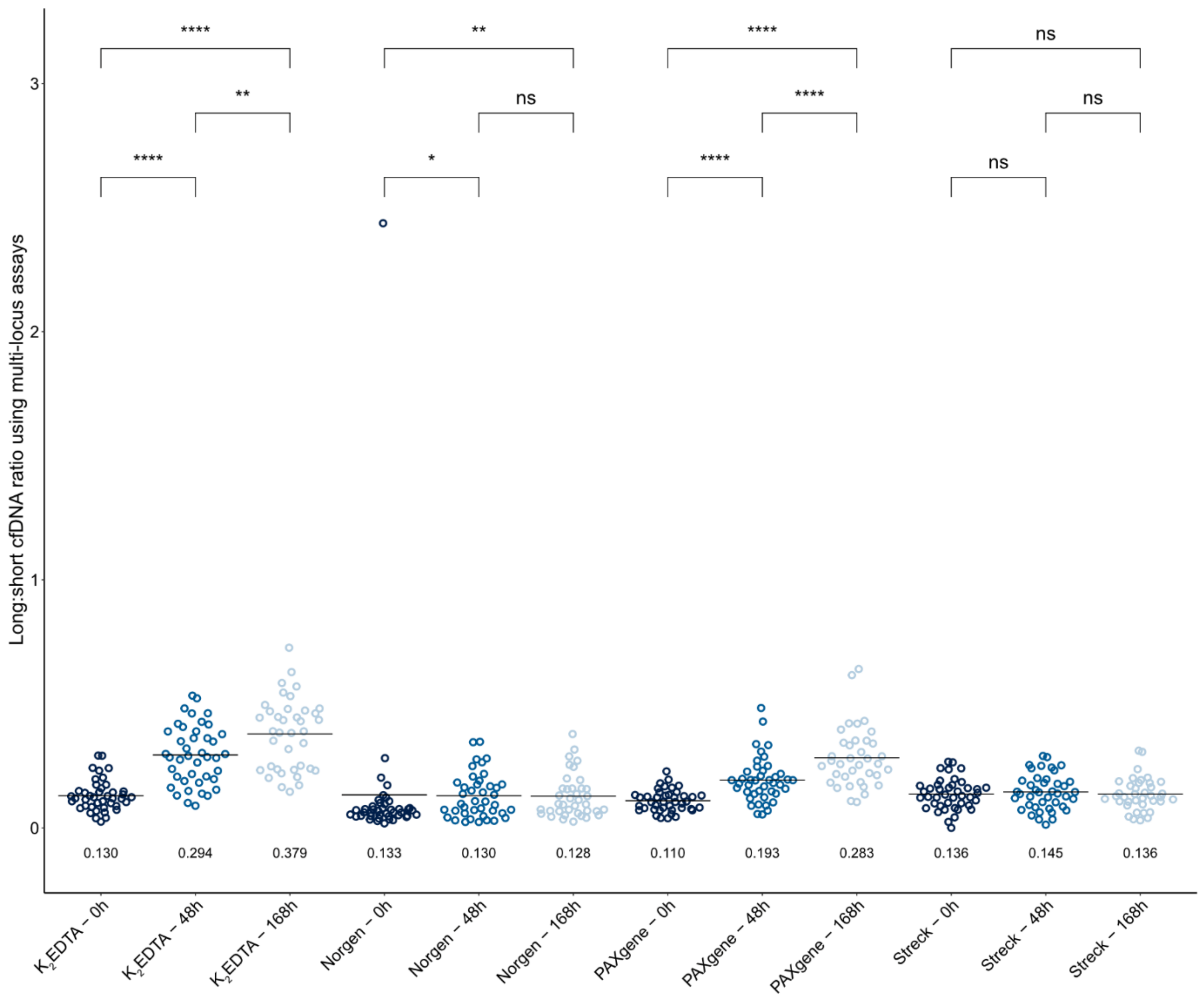

Supplementary Figure S6. Cellular DNA contamination assessed by multi-locus qPCR. The long:short cfDNA ratio using multi-locus assays is shown. The mean long:short cfDNA ratio is indicated by a bar and below the data points. Wilcoxon signed-ranked test was used, * p ≤0.05, ** p ≤0.01, *** p ≤0.001, **** p ≤0.0001 and ns, not significant.

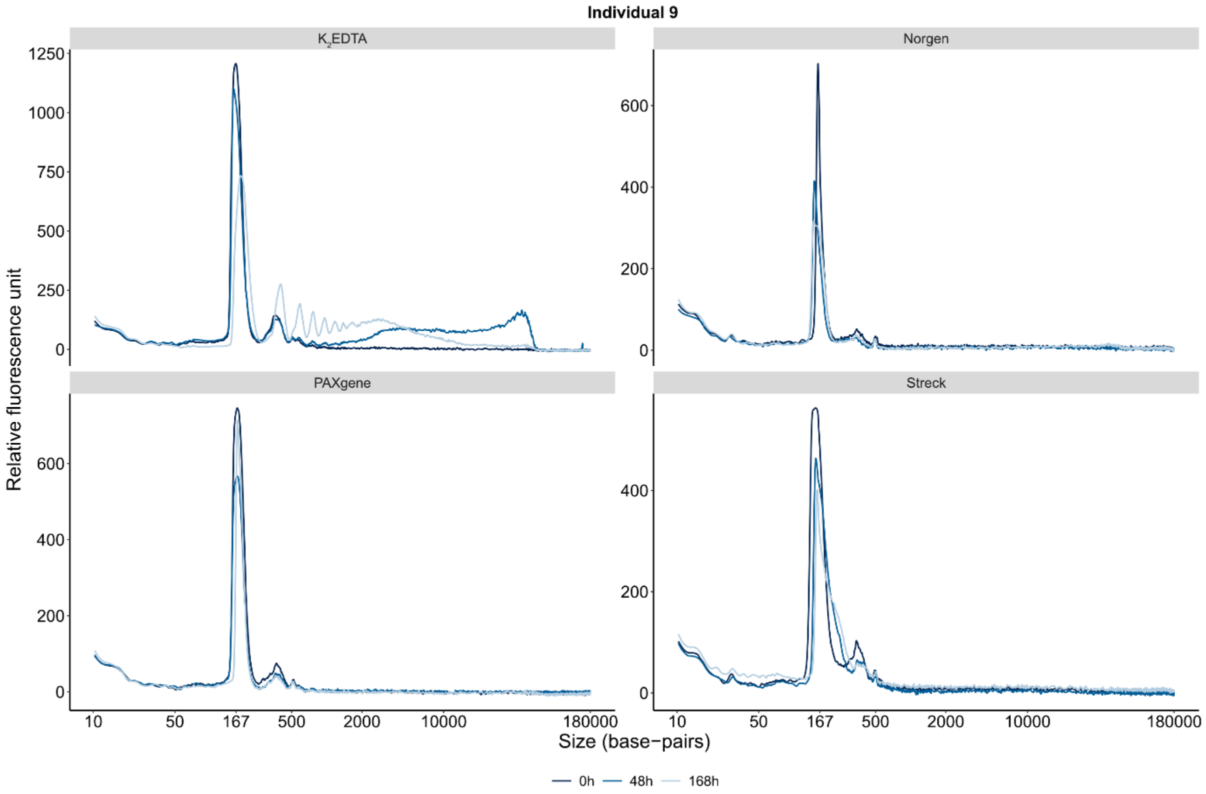
Individual 9
K2EDTA
Norgen
PAXgene
Streck
Relative fluorescence unit
10
50
167
500
2000
10000
180000
Size (base−pairs)
0h
48h
168h

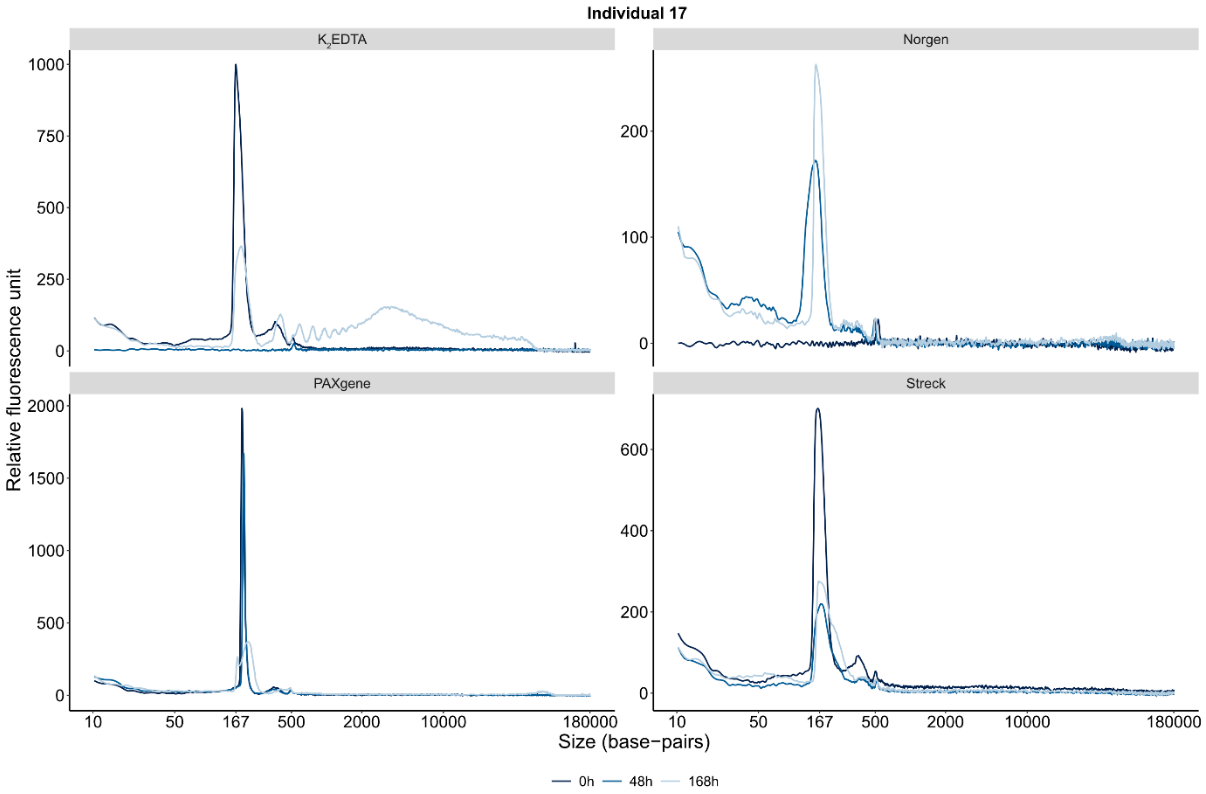
Individual 17
K2EDTA
Norgen
PAXgene
Streck
Relative fluorescence unit
10
50
167
500
2000
10000
180000
Size (base−pairs)
0h
48h
168h

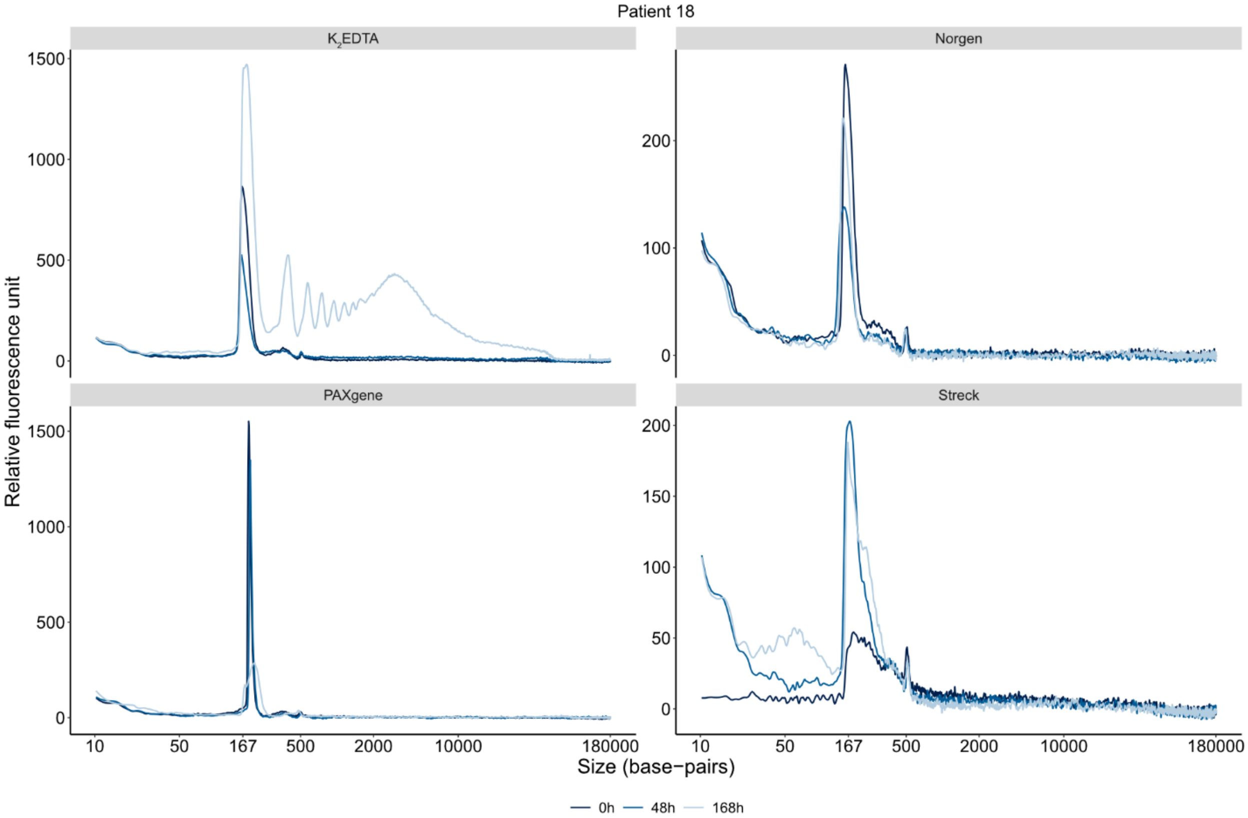

Patient 18
K2EDTA
Norgen
PAXgene
Streck
Relative fluorescence unit
Size (base−pairs)
0h
48h
168h

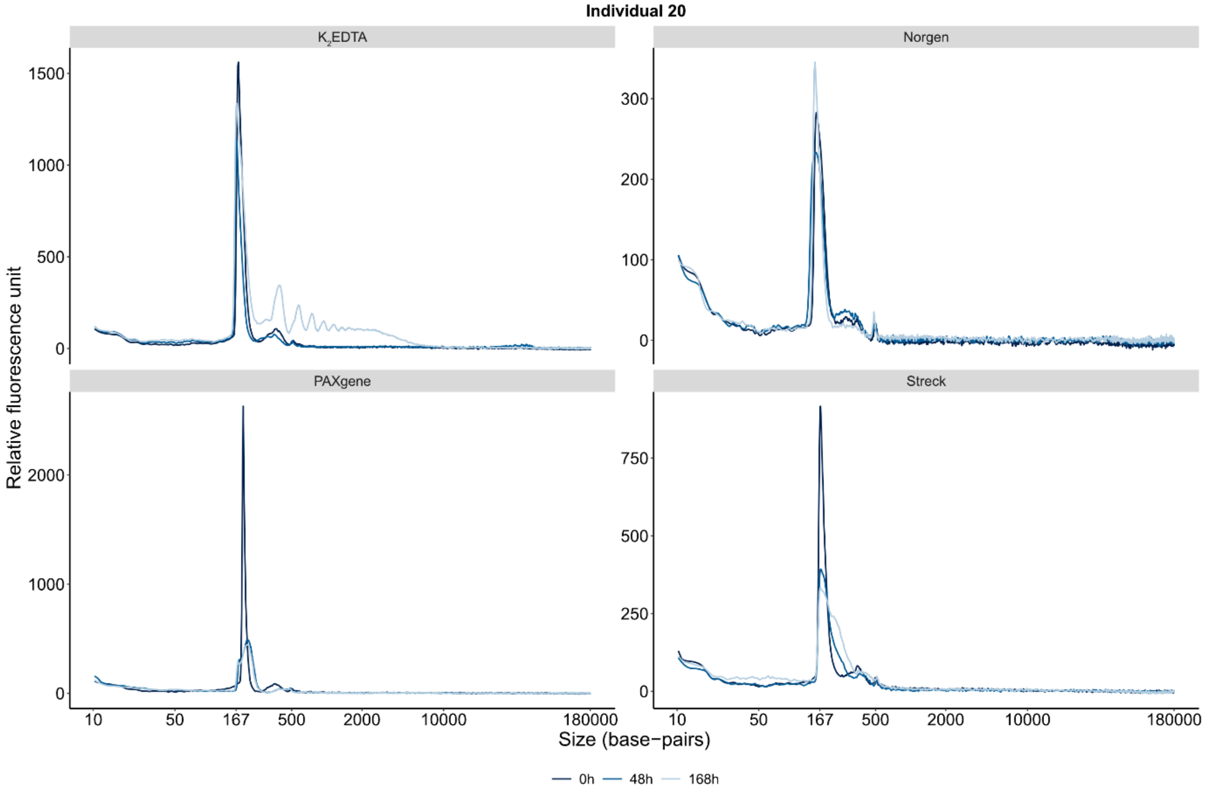

Individual 20
K2EDTA
Norgen
PAXgene
Streck
Relative fluorescence unit
Size (base−pairs)
0h
48h
168h

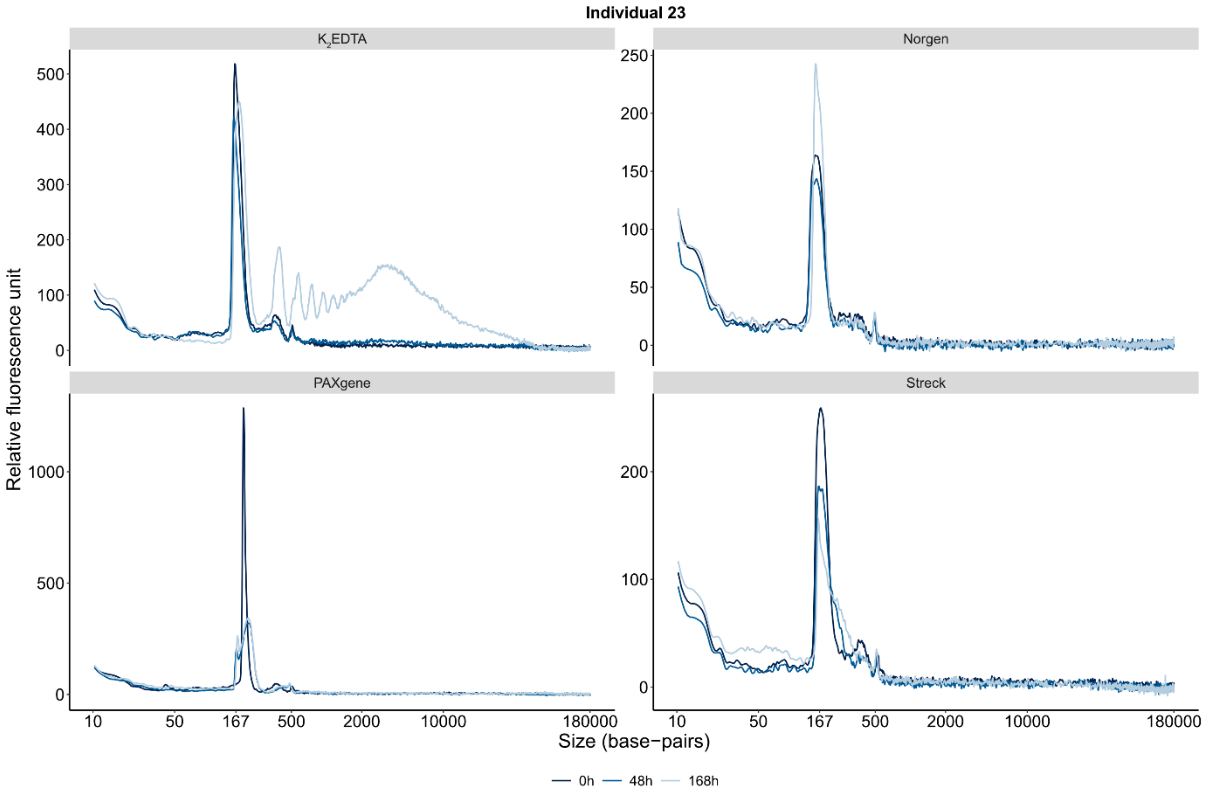

Supplementary Figure S7. Cellular DNA contamination assessed by parallel capillary electrophoresis. Electropherograms for K_2EDTA, Norgen, PAXgene and Streck tubes with plasma isolation after 0, 48 and 168 hours. Data are shown for individuals 9, 17, 18, 20 and 23.

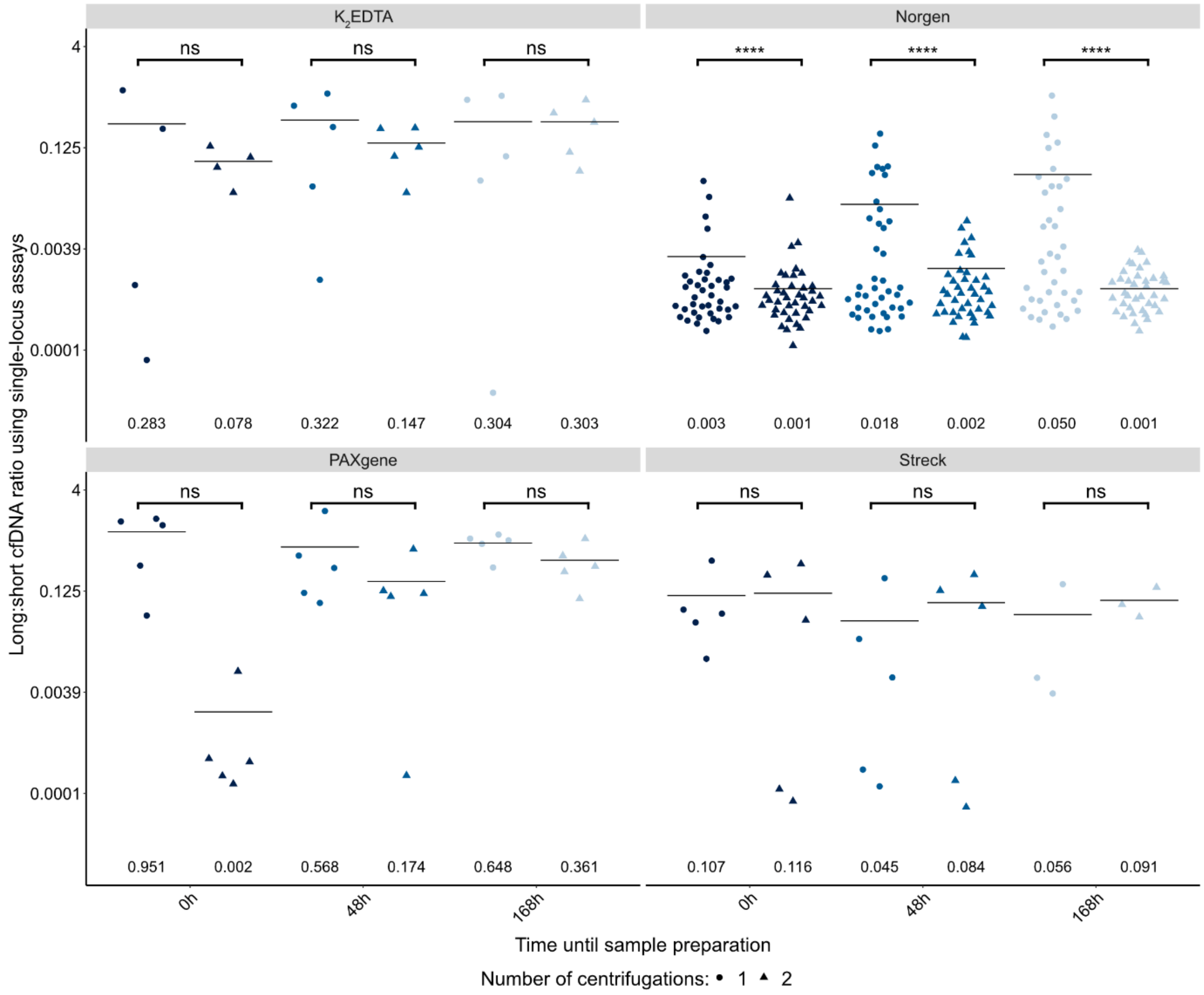

Supplementary Figure S8. Cellular DNA contamination in single and double centrifugated plasma. The long:short cfDNA ratio using single-locus qPCR is shown. The mean long:short cfDNA ratio is indicated by a bar and below the data points. Wilcoxon signed-ranked test was used, **** $p \leq 0.0001$, n.s., not significant.

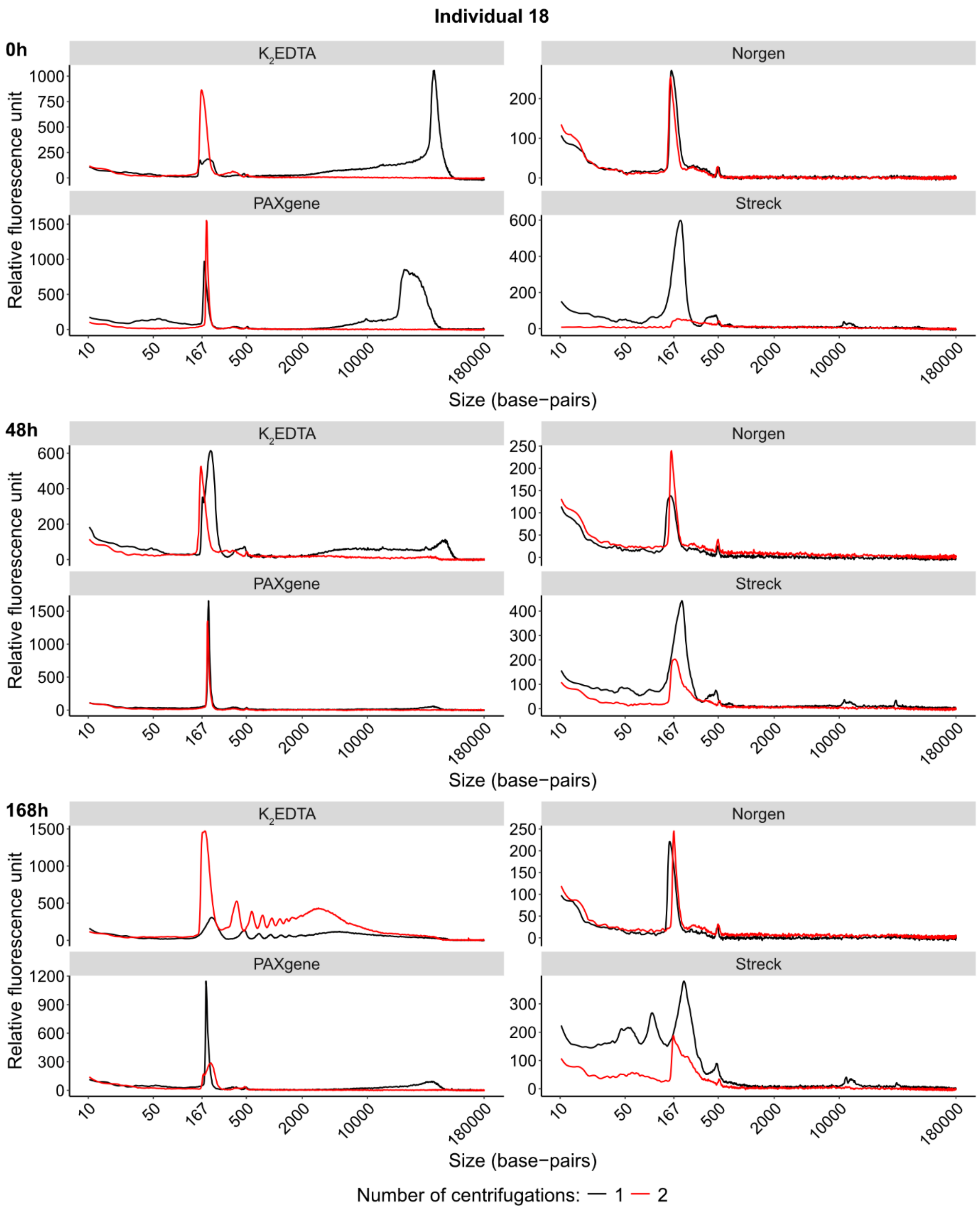

Individual 18
0h
48h
168h
K_2EDTA
Norgen
PAXgene
Streck
Relative fluorescence unit
Size (base−pairs)
10
50
167
500
2000
10000
180000
Number of centrifugations: 1 2

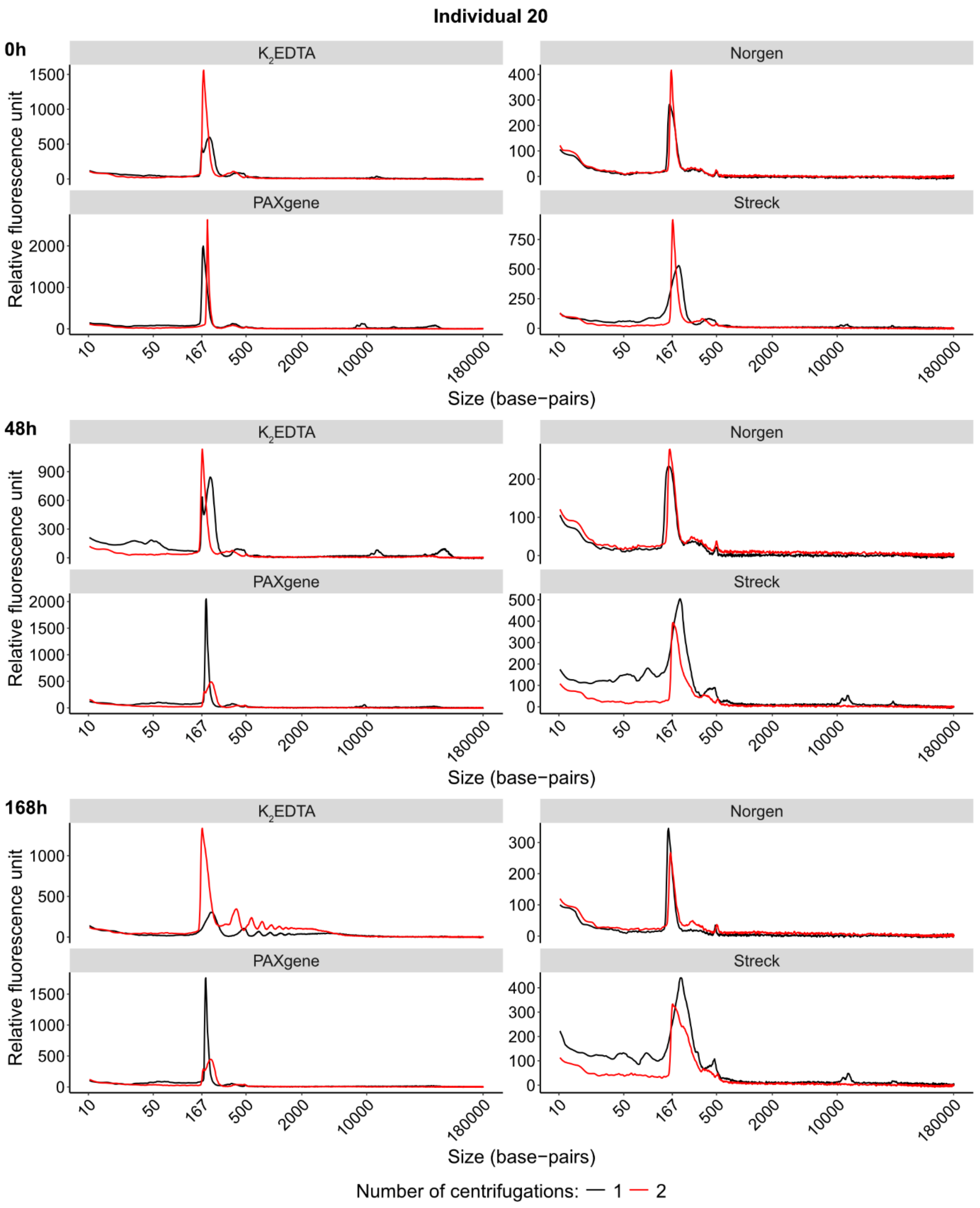

Individual 20
0h
48h
168h
K_2EDTA
Norgen
PAXgene
Streck
Relative fluorescence unit
Size (base−pairs)
10
50
167
500
2000
10000
180000
Number of centrifugations: 1 2

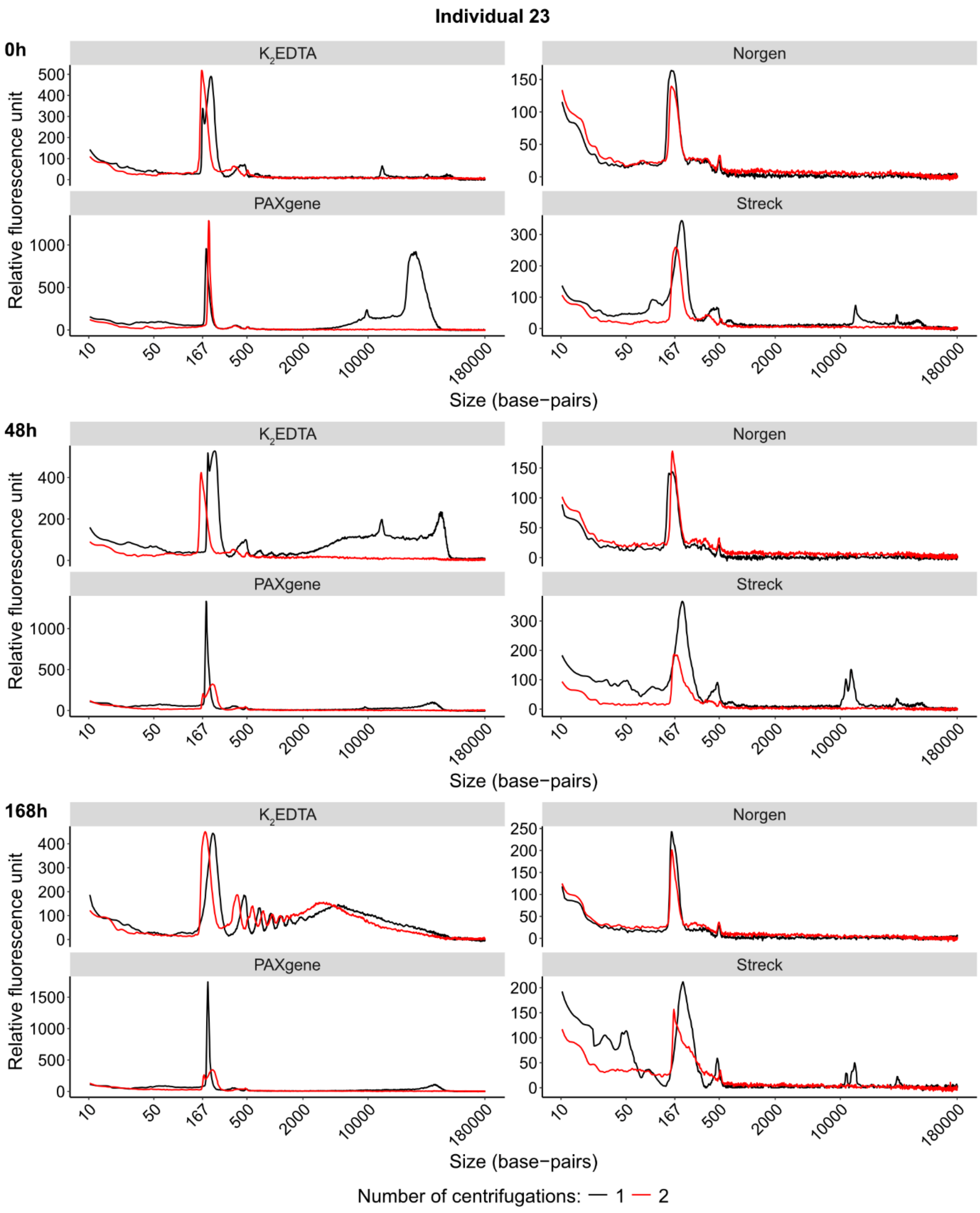

Supplementary Figure S9. Parallel capillary electrophoresis analysis of single and double centrifugated plasma. Electrophorograms for individuals 18, 20 and 23 are shown.
